# Tephra-mediated manganese cycling shapes coral responses to coastal sedimentation

**DOI:** 10.1038/s41598-026-38388-9

**Published:** 2026-02-04

**Authors:** Frank Förster, Christine Ferrier-Pagès, Allan Fries, Erouscilla Joseph, Tom E. Sheldrake

**Affiliations:** 1 Department of Earth Sciences, University of Geneva, Genève, Switzerland; 2 Ecophysiology Team, Centre Scientifique de Monaco, Monaco, Monaco; 3 Seismic Research Centre, The University of the West Indies, St. Augustine, Trinidad and Tobago

**Keywords:** Climate sciences, Ecology, Ecology, Environmental sciences, Ocean sciences

## Abstract

Terrestrial runoff from tropical volcanic islands impacts coral reefs by increasing turbidity and sedimentation. During explosive volcanic eruptions, large amounts of fragmented volcanogenic rock (tephra) are deposited, exacerbating sediment runoff for long periods of time. Nevertheless, tephra is an important, yet underestimated, source of the essential trace metal manganese (Mn), which promotes coral photosynthesis. Here, we show Mn leached from pristine and remobilised tephra increases resilience to sedimentation stress. Using coral culture experiments, microcolonies of *Stylophora pistillata* exposed to four tephra samples all showed rapid and sustained increases in photosynthetic efficiency (Φ_PSII_, rETR, P_n_ and P_gross_), even under reduced light conditions. Photosynthetic efficiency is logarithmically correlated to seawater Mn concentration, with large increases at values < 3 µg Mn L^− 1^, and negligible changes at values > 10 µg Mn L^− 1^. Tephra exposure has a crucial role in coastal Mn cycling and potentially benefits stressed corals following environmental disturbances.

**Supplementary Information:**

The online version contains supplementary material available at 10.1038/s41598-026-38388-9.

## Introduction

Explosive volcanic eruptions influence coastal marine environments over short to long timescales through the deposition and subsequent remobilisation of tephra, a collective term used to describe all rock fragments ejected in the atmosphere by volcanic eruptions. In the weeks, months, and years following the eruption, tephra deposits are progressively remobilised by wind- and water-driven erosion^1–3^. The extent and duration of the post-eruptive tephra transport depend largely on the nature of the volcanic impact, including the type of volcanic hazard (e.g. ash fallout, pyroclastic flow, and lahars) and tephra characteristics such as total erupted volume, and have varying temporal characteristics^4^. Heavy rainfall can dramatically increase tephra discharge^5^, which further accelerates deposit erosion and often triggers destructive debris flows^6^. Volcanic river systems may experience up to a 100-fold increase in persistent sediment transport, which can last for decades^4^. This sustained sediment influx enhances the load of tephra in coastal waters.

The increased sediment input substantially elevates coastal seawater turbidity^7^ and decreases surface irradiance, which has critical implications for near coral reef ecosystems. Scleractinian corals, which are the main architects of coral reefs, are sensitive to changes in seawater clarity, as they live in symbiosis with Symbiodiniaceae algae^8^, which require light for photosynthesis^9^. Traditionally, it is assumed that increased turbidity has a negative impact on coral photosynthesis by reducing the photosynthetically active radiation (PAR)^10^. This can lead to reduced coral growth^11^, coral bleaching, the stress-induced expulsion of the Symbiodiniaceae, and ultimately coral mortality^9^. In addition to elevated turbidity, sediment loading can also introduce pollutants, including macronutrients such as nitrate and phosphate, which can further disrupt the reef ecosystem^12^. Elevated sediment loads have therefore been associated with a decline in larval settlement, photosynthetic efficiency^10^, and fertilization success^13^. Despite these challenges, several studies have documented high coral cover on turbid reefs^14–17^ and enhanced resistance during heatwaves^17–19^, with coral species able to photoacclimate to low-light conditions^20^. Some ancient reefs also accreted despite sustained exposure to turbidity and sedimentation^21,22^.

Corals exposed to sediment input (e.g., from tephra runoff) can benefit from metal enrichment^23^. Indeed, geogenic particles, such as desert dust and volcanic ash, are a known source of essential metals such as Mn for coral reefs in oligotrophic tropical waters^23–26^. The Mn input from subaerial volcanism is estimated to be of a similar order of magnitude to the aeolian dust inputs, implying the ash-exposure Mn release is an important, but yet underappreciated source to global biogeochemical cycles^27^. As an essential bioelement, Mn, in its bioavailable Mn^2+^ form, is quantitatively the second most important trace metal for all photosynthetic organisms, including Symbiodiniaceae^8^ which live in a mutualistic endosymbiosis with their reef-building coral hosts. Mn is involved in chlorophyll biosynthesis *via* the isoprenoid biosynthetic pathway^28^ and in the light-dependent reactions of photosynthesis^29^. It is crucial for oxygen production, serving as a cofactor in the oxygen-evolving complex (OEC) of photosystem II^30^. In addition to its function in algal photosynthesis, it is also a cofactor in antioxidant enzymes such as Mn superoxide dismutase^31^. In laboratory settings, exposure to desert dust and volcanic ash led to elevated Mn accumulation in tissue and symbionts of exposed coral microcolonies^23,25,26^. All of these studies also reported increases in the photosynthetic efficiency and chlorophyll concentrations of the Symbiodiniaceae, as well as in net and gross photosynthesis rates (P_n_ and P_gross_), a trend also observed with Mn supplementation alone^32,33^. Specifically, Mn addition (4.1 ± 0.75 µg Mn L^− 1^) was linked to significant improvements in the photosynthetic efficiency (P_n_, P_gross_, and rETR) in temperate *S. pistillata* nubbins (26 °C)^33^. Finally, Mn can enhance coral resistance to heat-stress induced bleaching^33,34^. In all these studies, Mn enrichment was limited to few µg L^− 1^. At concentrations > 1000 µg Mn L^− 1^, far exceeding open ocean (0.004–0.27 µg Mn L^− 1^) and coastal waters (0.082–0.38 µg Mn L^− 1^) Mn levels^35^, dissolved Mn becomes toxic to marine biota^36^. Tissue sloughing, which describes the disconnection of coral tissue from the skeleton without loss of endosymbionts^37^, was observed at a 48-hour median effect concentration (EC_50_) of 700–2596 µg Mn L^− 1^ for adult branching corals (*Acropora spathulata*,* Acropora muricata*,* Acropora millepora* and *S. pistillata*)^35,37–39^, which corresponds to a chronic toxicity of 70–259 µg Mn L^− 1^ assuming a default acute-to-chronic ratio of 10. Golding, Binet^35^ did not observe chronic toxicity (after 14 days) up to 1090 µg Mn L^− 1^ for *Acropora millepora*, highlighting a species-specific tolerance to high Mn concentrations. Additionally, the coral age also plays a role in the Mn sensitivity, as adult corals are more susceptible to Mn toxicity than coral early-life stages^37^.

To gain further knowledge on how different tephra, collected from various depositional settings, influence seawater Mn concentrations, we conducted several three-week experiments using the scleractinian coral *S. pistillata*, with daily additions (1 g L^− 1^) of tephra sampled from around La Soufrière volcano on St. Vincent (Eastern Caribbean). During the experiments, we assessed the physiological response of corals to understand the impacts of tephra deposition on coral health. Our findings highlight the role of volcanogenic sediments in Mn supplementation and contribute to a broader understanding of Mn fluxes in coastal waters. By integrating ecophysiological measurements with geochemical data of the tephra and seawater, we provide new insights into coral reef dynamics to show that Mn is a key element in providing resistance to stress associated with increased sediment input around volcanic islands.

## Methods

### Tephra samples

**Fig. 1:**
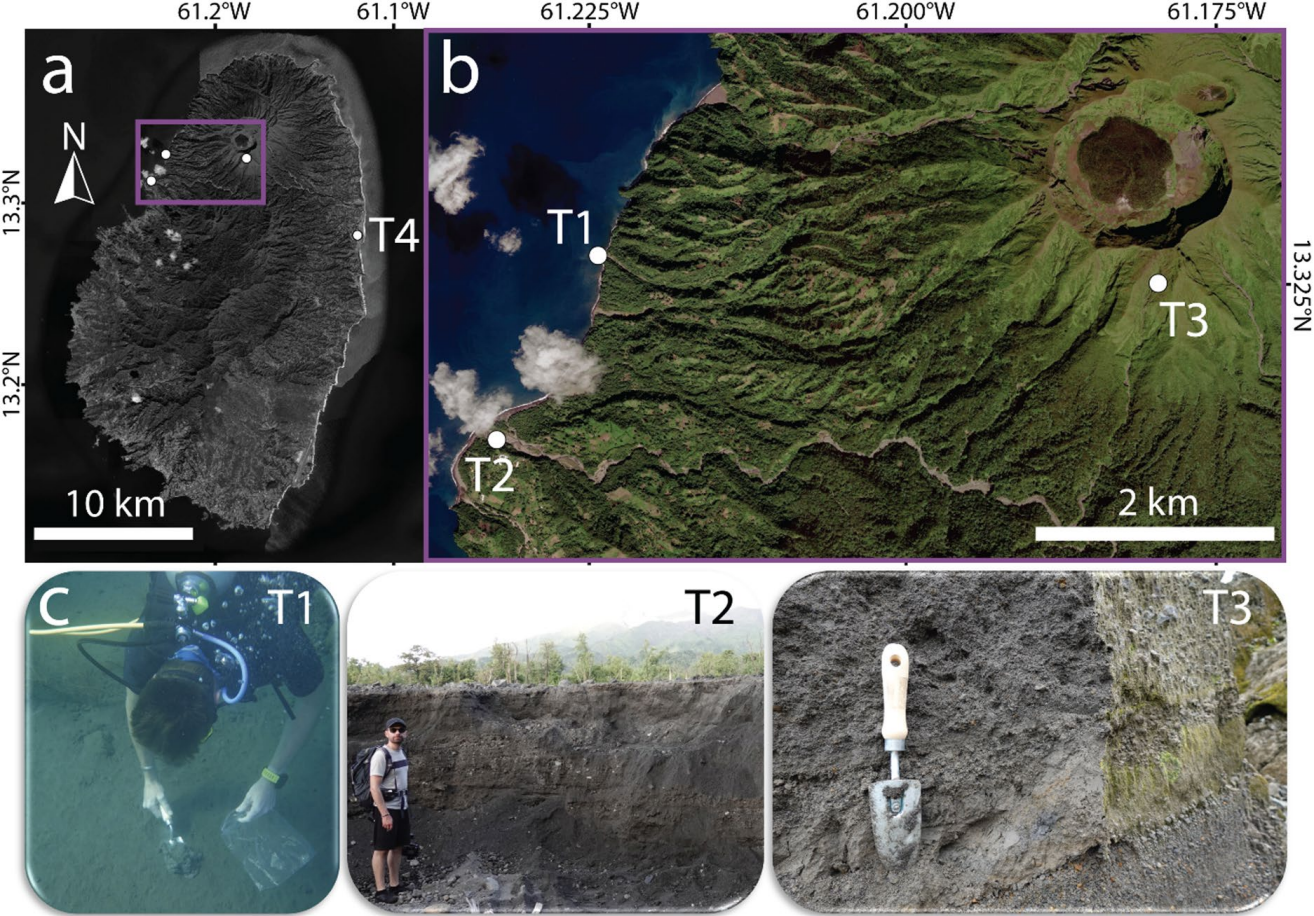
Map of **(a)** St. Vincent (Eastern Caribbean) and the highlighted **(b)** sampling locations of T1-T3 on the northwestern part of the island. **(c)** Photographs of the sampling locations for tephra samples T1, T2 and T3 with persons shown for scale. Consent for publication was obtained. Tephra was retrieved when the picture was taken (for T1) or soon after the picture was taken (for T2 and T3). Dates of collection are presented in Table 1. The map was created using QGIS software v. 3.34^44^ and the ESRI World Imagery. Sources: Esri, DigitalGlobe, GeoEye, i-cubed, USDA FSA, USGS, AEX, Getmapping, Aerogrid, IGN, IGP, swisstopo, and the GIS User Community https://services.arcgisonline.com/arcgis/rest/services/World_Imagery/MapServer/tile/{z}/{y}/{x}.

Four tephra samples (each ≈ 1 kg) were collected on the volcanic island of St. Vincent during and after the explosive activity of La Soufrière volcano on 9–22 April 2021 (Tab. 1; Fig. 1). A bulk volume of approximately 120 × 10^6^ m^3^ of tephra was emitted during this eruption, which produced volcanic plumes rising to 23 km in the atmosphere^40^. Tephra deposits are stratified and characterised by numerous lapilli- and ash-rich layers, comprising tephra originating from both pyroclastic density currents (PDCs) and fallout^41^. The bulk rock composition of the emitted tephra exhibits SiO_2_ contents ranging from 52.8 to 55.4 wt%, classifying them as basaltic andesite, with a MnO content of ≈ 0.2 wt% in both the bulk and measured in the glassy groundmass^42^. Three of the four tephra samples (T1 – T3) used in this study were collected during a field campaign in February/March 2024, while one pristine ash sample (T4) was collected immediately after deposition on 11.04.2021 in Georgetown (eastern part of St. Vincent, Fig. 1a). T1 and T2 represent remobilised tephra, whereas T3 and T4 represent tephra collected in situ following deposition. Tephra samples were stored in plastic zipper bags and transported to the University of Geneva for further analysis.

**Table 1:** Tephra sample information.

Tephra sample	Date of collection	Location of collection	Latitude	Longitude	Further information
T1	29.02.2024	Morne Ronde River delta	13.32740	−61.22398	marine deposit
T2	27.02.2024	Quarry in Wallibou River	13.31290	−61.23194	remobilized lahar deposit
T3	02.03.2024	Summit crater – upper SE flank	13.32521	−61.17996	In situ PDC deposit
T4	11.04.2021	Police station, Georgetown	13.28098	−61.11736	pristine ash

Tephra samples were dried in an oven (40 °C) for a week before 4-mm sieving. Sieved samples were ground to a homogenous particle size using a Pulverisette 2 grinder (FRITSCH^®^) equipped with an agate mortar and pestle. This was to control for the effect of particle size^43^ during coral culture experiments. In addition to their use in the coral culture experiments, aliquots of the tephra samples were analysed for their: (i) particle size distribution using Bettersizer S3 Plus (Bettersize^®^); (ii) bulk mineralogical composition using X-ray diffraction (XRD); (iii) the bulk chemical composition using X-ray fluorescence spectroscopy (XRF); (iv) amount of volcanic glass using a Scanning Electron Microscope (SEM); and (v) the chemical composition of the volcanic glass using electron microprobe (EPMA).

#### Particle size distribution

A total of three aliquots per tephra sample were analysed with the Bettersizer S3 Plus Laser Particle Size and Shape Analyzer at the University of Geneva. Particle size distributions were obtained through laser diffraction and are characterised by the median particle size [µm] and sorting coefficient (σ)^45^.

#### X-ray diffraction

The bulk mineralogical composition of the tephra samples was determined by X-ray powder diffraction (XRD). XRD measurements were performed in reflection mode (Bragg-Brentano geometry) on a Panalytical^®^ Empyrean diffractometer equipped with a copper anode (Cu-Kα_1_). Data were recorded in the continuous-scan mode at 45 kV and 40 mA between 5 and 80° (2θ angle) with a step size of 0.013°. XRD data are presented as diffractograms, which were evaluated using Diffrac.Eva software (v.6.1) for peak finding and matching with XRD patterns from a database. XRD patterns of reference compounds are listed in the International Centre for Diffraction Data’s (ICDD^®^) Powder Diffraction File (PDF^®^)−5 + 2024.

#### XRF bulk tephra analysis on fused beads

Major and minor element compositions of the bulk tephra samples were measured by X-ray fluorescence (XRF) analysis on prepared XRF glass beads. Sample preparation involved weighing 1.2 g of calcinated tephra (heated at 1050 °C for 3 h) and 6 g of lithium tetraborate flux (Spectromelt A10 Merck) using a Mettler Toledo AG245 balance (accuracy ± 0.01 mg). The mixture was then fused to a glass bead at 1200 °C using a Claisse Eagon 2 fusion machine (Malvern Panalytical). Glass beads (one per tephra) were then analysed with the Panalytical Axios^mAX^ XRF spectrometer at the University of Lausanne. The obtained values from the CRM BHVO-2 deviate by < 1% from GeoReM preferred values (Tab. S1). Major and minor element compositions of the bulk tephra samples are expressed as oxides (in wt%; Tab. S2).

#### SEM-EDS for quantification of volcanic glass content

The amount of volcanic glass in the tephra samples was quantified following the procedure presented in Horwell, Sparks^46^ (Tab. S3). Approximately 50 random particles per tephra sample were analysed for their major element composition using an SEM-Energy Dispersive X-ray Spectroscopy (EDS) system consisting of a JEOL JSM7001F SEM with energy-dispersive analyser EDS JED2300 in the Department of Earth Sciences, University of Geneva. The sample preparation included the mounting of finely dispersed tephra on sticky carbon tape attached to aluminium stubs and carbon coating. Particles were analysed within randomly selected fields of view (Fig. S1a). After completing the analysis of particles in a given field, the field of view was changed, and the process was repeated until approximately 50 particles had been examined. The chemical composition was determined for particles in various magnifications using an accelerating voltage of 10 kV, a probe current of 1 nA and a working distance of 10 mm. Mineral and glass identification was based on the comparison of major elemental compositions, expressed as oxides, with reference data for individual phases from Weber, Blundy^42^. Representative EDS spectra for the identified minerals and glass can be found in Fig. S1b. Due to the relatively small number of particles per sample (*n* ≈ 50), phase proportions are reported as percentages with accompanying 95% confidence intervals (CIs). The CI is calculated as 1.96 x the Standard Error (SE), where SE is expressed as

$$\:SE\:=\sqrt{\frac{p\:(1-p)}{n}}$$.

with $\:p$ representing the proportion, and $\:n$ the sample size (i.e., here the total number of particles analysed).

#### EPMA analysis of volcanic glass

50 electron microprobe (EPMA) measurements were conducted on randomly selected glass particles from each of the tephra samples T1 – T4. Volcanic glass was analysed for its major and minor elements (Si, Al, Fe, Ca, Mg, Na, Ti, K, P, and Mn) using a Hyperprobe-JXA-IHP200F EPMA (Tab. S4), with wavelength dispersive spectrometers (WDS) and a 15 kV accelerating voltage at the University of Geneva. Measurements were performed using a beam current of 3 nA, accompanied by a spot size of 2 μm. The spot size was selected based on the microlitic texture of the glass particles in the tephra samples^42^. The analyses were calibrated against various natural mineral and glass standards: glass Smithsonian A99 for Si and Al; fayalite for Fe; wollastonite for Ca; olivine for Mg; albite for Na; manganese titanite for Ti and Mn; orthoclase for K; apatite for P. Peak and background counting times for all elements were set at 30 s and 15 s, respectively, except for P and Mn (60s and 30 s, respectively), and for Na and K (20s and 10 s, respectively). The reduced times for Na and K were applied to minimize alkali loss.

Quality control (accuracy and precision) was assessed on replicate measurements (*n* = 4) of the Smithsonian basaltic glass VG2 (Tab. S5). Precision, expressed as two times the relative standard deviation, was < 20%, except for K (27.98%) and P (43.91%), and measured values deviated < 5% from (GeoReM preferred) consensus values, except for Mn (21.14%) (Tab. S5). The chemical composition of volcanic glass in the tephra samples is expressed as oxides (in wt%).

Data evaluation included multiple “data cleaning“ steps: (i) removal of all analyses with totals < 95 wt.% and > 105 wt.%, (ii) removal of analyses with SiO_2_ content < 60 wt.%, as these likely represent mixed signals or sampled plagioclase according to Weber, Blundy^42^, (iii) identification and removal of outliers using a multivariant outlier test on normalized and log-transformed data (described in Sect. 2.5).

### Coral culture experiments

Coral culture experiments were conducted at the Centre Scientifique de Monaco using lab-grown microcolonies of the scleractinian coral *S. pistillata.* This model coral species was selected because of its frequent use in laboratory stress studies^47^, and its well characterised physiology, including responses to various stressors such as metals^33,48–51^. Coral nubbins (3–4 cm in size) were cut from several large parent colonies four weeks before the experiments and attached to a nylon fishing thread. These nubbins were given time to heal for four weeks, during which they were fed twice a week with *Artemia salina* nauplii, the same food the parent colony was fed and the coral is accustomed to. During this time, they were kept in aquaria maintained at 26 °C and receiving a PAR of 200 µmol photons m^− 2^ s^− 1^ with a photoperiod of 12 h of light. Aquaria were cleaned once a week to avoid the development of algae.

**Fig. 2:**
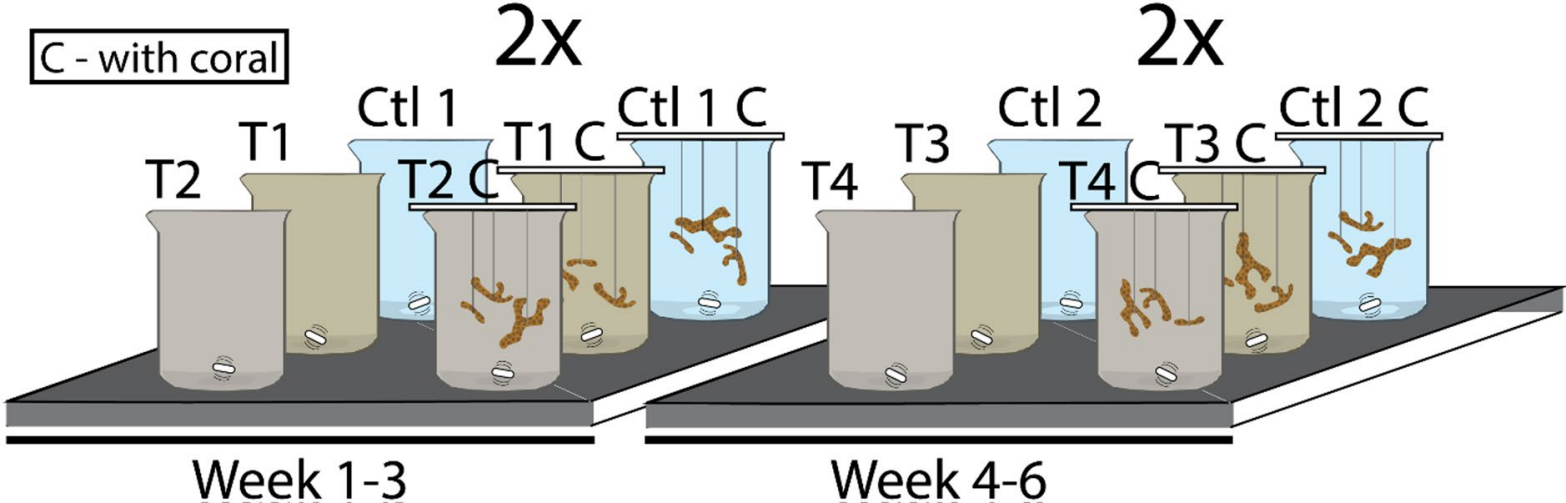
Experimental setup. The experiment was conducted in replication, and in two phases: (I) Week 1–3 and (II) Week 4–6. Each experiment phase comprises 12 beakers, half equipped with 3 nubbins of *S. pistillata* in each beaker (beakers labelled with “C”). “Ctl” indicates control condition, while “T” represents tephra conditions.

The experimental setup comprised a set of twelve glass beakers (1 L) placed on magnetic stirring plates (Fig. 2). Four beakers were assigned per condition, half contained only seawater (without corals), while the other half included three nubbins of *S. pistillata* originating from different mother colonies. The experiment was conducted in two phases: In the first three weeks, one control condition (Ctl1) and two experimental conditions (T1 and T2) were used, while in weeks 4–6, a second control (Ctl2) and two additional experimental conditions (T3 and T4) were tested. Feeding was stopped before the experiments, to not interfere with the metals released by the tephra. Each condition was replicated so that there were two beakers per condition and thus twelve beakers at any one time.

Each morning (Monday-Friday), 1 L of 0.45-µm filtered seawater was added to each beaker and heated to 26 °C using a water bath. The irradiance was kept identical to the culture conditions. Once the water temperature stabilized at 26 °C, coral nubbins were introduced into designated beakers with 1 g of tephra added at 9 am, to achieve a tephra-to-seawater concentration of 1:1 (g L^− 1^). This tephra concentration was selected based on its common use in ash-water leachate studies^52,53^ and to allow comparability with previous research. In each beaker, a magnetic stirrer ensured constant temperature and resuspension of tephra throughout the beaker. Changes in irradiance and turbidity following tephra addition were quantified through multiple daily light measurements in the beakers without corals at various time points (between 0 and 9 h after tephra introduction) using a ULM-500 Light Meter & Logger (Walz GmbH^®^, Effeltrich, Germany) connected to a quantum sensor (LI-193). After 8–9 h of tephra exposure, coral nubbins were transferred to fully controlled 30 L regeneration aquaria, where they remained overnight before being reintroduced into the experimental beakers the following day. This regeneration step was necessary because continuous evaporation in the 1 L beakers would lead to increasing seawater salinity. The regeneration aquaria were maintained at the same light and temperature conditions as the experimental setup. They received a constant supply of seawater, which was mixed using a water pump (Newa, Loreggia, Italy) to ensure temperature homogenisation and was cleaned weekly to prevent filamentous algal growth. The 1 L beakers were rinsed daily with tap water, dried, and reused the next morning.

To quantify tephra-supplied Mn concentrations in seawater, 10–15 mL of seawater was sampled once per week (on Mondays, at the end of the day) from each beaker (*n* = 12) using a 50 mL polypropylene (PP) syringe, equipped with a 0.22 μm polyethersulfone (PES) syringe filter (Fisherbrand^®^). Both were rinsed three times with 0.45-µm filtered seawater before usage. The collected solution was transferred to metal-free PP tubes, acidified to 0.5 M HNO_3_ with ultrapure nitric acid (NORMATOM^®^), and stored at 4 °C. After the three weeks of exposure experiments, nubbins were stored at −20 °C for further analysis.

### ICP-MS on seawater samples

Total dissolved Mn concentrations in the sampled seawater were determined using single-quadrupole inductively coupled plasma mass spectrometry (ICP-MS, Agilent 7700) at the University of Geneva (Switzerland). Before analysis, samples were diluted 100 times with 0.5 M HNO_3_ (NORMATOM^®^). Measurements were conducted over two separate days. Mn concentrations were quantified using a nine-point calibration curve, prepared shortly before analysis. Calibration standards were created by diluting the ICP multi-element standard solution IV (Certipur^®^) with 0.5 M HNO_3,_ aiming at Mn concentrations of 0, 0.1, 0.5, 1, 5, 10, 50, 100, and 300 µg L^− 1^. Mn was measured as ^55^Mn in helium mode with an integration time of 1 s. Scandium (50 µg L^− 1^) was used as an internal standard to assess analytical drift. Accuracy and precision were assessed against a matrix-matched certified seawater reference material (CRM-SW-250, hps^®^), with a recovery of 101.06% (our value/official value) and precision of 5.15% relative standard deviation (*n* = 8). The detection limit (LOD) was determined to be ≈ 0.2 µg L^− 1^ on both days. Mn concentrations are reported in µg Mn L^− 1^ and presented in Table S6.

### Physiological measurements

#### Chlorophyll fluorescence measurements

Twice a week (Mondays and Thursdays), the chlorophyll fluorescence of photosystem II (PSII) was measured on all nubbins (*n* = 6 per condition) using a Pulse-Amplitude-Modulated (PAM) fluorometer (DIVING-PAM, Walz^®^, Germany). The effective quantum yield of photosystem II (Φ_PSII_), measured at 200 µmol photons m^− 2^ s^− 1^, indicates how effectively light energy is used for symbiont photosynthesis, reflecting the coral’s photophysiological state. Monitoring the photosynthetic performance of light-adapted corals is crucial, as changes in Φ_PSII_ are linked to stress^54^. Φ_PSII_ is calculated according to Genty, Briantais^55^ as

$$\:{\varPhi\:}_{PSII}=\:\frac{{{F}^{{\prime\:}}}_{m}-{F}_{0}}{{{F}^{{\prime\:}}}_{m}}$$.

with F_0_ and F’_m,_ respectively, minimum and maximum fluorescence yields at 200 µmol photons m^− 2^ s^− 1^. Rapid light curves (RLCs) were measured at the end of the experiment on all nubbins, which were dark-adapted for 15 min before analysis according to Hoogenboom, Campbell^56^. The nubbins were exposed to increasing light intensities for 10 s intervals across eleven steps, ranging from 0 to 1956 µmol photons m^− 2^ s^− 1^ (0, 10, 17, 26, 41, 74, 130, 220, 664, 1032, 1956 µmol photons m^− 2^ s^− 1^). The RLC-derived photochemical parameters (relative electron transport rate (rETR) and non-photochemical quenching (NPQ)) were plotted as a function of the photosynthetically active radiation (PAR [µmol photons m^− 2^ s^− 1^]). The data are visualized by using the WinControl_v.3.29 program (Walz GmbH^®^, Effeltrich, Germany).

#### Skeletal growth rate

The growth rate of all nubbins (*n* = 6 per condition) was monitored throughout the entire experimental period. Nubbin length and weight were measured weekly to assess linear extension rates and skeletal mass gain. The linear length was measured using a digimatic caliper (Mitutoyo^®^) and weight was determined using the buoyant weight technique^57^ with a Mettler Toledo XP205 balance (accuracy ± 0.01 mg). Data are presented as total value [mm or g] or as percentage changes [%] in linear extension and weight gain relative to the initial measurements.

#### Photosynthesis and respiration rates

Rates of respiration (R), gross (P_gross_) and net photosynthesis (P_n_) were measured on two nubbins per condition after three weeks of exposure to tephra. Each nubbin was placed in Plexiglass^®^ chambers (60 mL) containing 0.45 μm-filtered seawater, maintained at the experimental temperature (26 °C) and continuously stirred using a magnetic stirrer. The oxygen concentration was continuously measured using a polymer optical fiber sensor (PreSens^®^) connected to an Oxy-4 fiber-optic oxygen meter (PreSens^®^) and recorded with the Oxy4v2-30fb software. P_n_ was measured under 200 µmol photons m^− 2^ s^− 1^ irradiance, while R was measured in the dark, both for approximately 45 min. P_gross_ was calculated as the sum of R and P_n_. Calibration was performed on seawater saturated with 100% O_2_ and 0% O_2_ (N_2_-saturated) at ambient air pressure. P_gross_, P_n_ and R rates were normalized to both the coral surface area and the total symbiont count, expressed as µmol O_2_ h^− 1^ cm^− 2^, or pmol O_2_ h^− 1^ symbiont cell^− 1^, respectively. Coral surfaces were measured using the single-dip wax technique^58^ on cleaned skeletons.

#### Symbiont density, protein holobiont content, and chlorophyll a and c_2_ content

Coral tissue and symbionts were separated from the skeleton using an airbrush with pressurized air and 0.45-µm filtered seawater. The extract containing both host tissue and symbionts was sequentially homogenised for 20 s using an Ultra-Turrax T25 (Janke & Kunkel (IKA)^®^). The 10–20 mL homogenate was then distributed into multiple sub-samples for the following measurements. Three 10 µL sub-samples were used to quantify dinoflagellate density using a LUNA FX7 automated Cell Counter (Logos Biosystems, South Korea). Another 500 µL subsample was used to determine the protein content using Bradford Assay (Thermo Scientific™ Protein Dosage Pierce™ Coomassie kit reference: 23200) according to Smith, Krohn^59^. A calibration curve was prepared with 2 mg mL^− 1^ BSA (Bovine Serum Albumin) with serial dilutions (0–2000 µg mL^− 1^). The subsample was then mixed *via* microplate vortexing with 0.5 M NaOH and the Pierce Coomassie Assay Reagent, and incubated at 60 °C for 30 min. Absorbance was measured at 595 nm using a Xenius^®^ SAFAS spectrophotometer. Protein content [mg] was normalized to skeletal surface area [cm^2^]. A 5 mL sub-sample was centrifuged at 5000 G for 10 min at 4 °C to separate host tissue and dinoflagellates. The supernatant was discarded and the concentrate containing symbionts was resuspended with 5 mL of pure Acetone. The mixture was then kept in the dark for 24 h at 4 °C, before centrifugation at 5000 G for 15 min at 4 °C. 300 µL of supernatant was added in triplicate in a quartz microplate and absorbance was measured at 630 and 663 nm using a Xenius^®^ SAFAS spectrophotometer. Chlorophyll a and c_2_ concentrations were calculated following equations by Jeffrey and Humphrey^60^ and Ritchie^61^, and normalized to the skeletal surface area and total symbiont number.

### Statistical evaluation

Statistical tests were performed on datasets that included six or more observations per condition to ensure statistical robustness. These datasets comprised the compiled Mn seawater measurements, light measurements, chlorophyll fluorescence measurements and the growth parameters. However, physiological parameters, such as symbiont density and total chlorophyll concentration, were excluded due to their small sample size (*n* = 2 per condition). Instead, these data are described qualitatively in the results section.

Statistical analyses were performed using RStudio Version 4.2.0^62^. Before applying appropriate statistical tests to compare experimental conditions, preliminary assessments were conducted. The Shapiro-Wilk test was used to check for normal distribution within each condition, while Levene’s test assessed variance homogeneity across conditions. Since most datasets followed a normal distribution and parametric tests offer greater statistical power, parametric methods were preferred for quantifying differences between conditions. For comparisons, T1 and T2 were always analyzed against Ctl1, while T3 and T4 were compared against Ctl2. A p-value < 0.05 indicates a statistically significant difference. The statistical results are provided in the data repository file.

EPMA data of volcanic glass (*n* = 50 per tephra sample) were investigated for outliers using a multivariant outlier detection method based on robust covariance and Mahalanobis distance (R packages provided by Todorov^63^, van den Boogaart^64^. Prior to analysis, data were normalized to 100% and subjected to isometric log-ratio transformation. Mahalanobis distances were then calculated, and the top 2.5% points with the most extreme distances were identified as outliers and removed from subsequent evaluation.

Non-linear curve fits between two independent parameters (i.e., seawater Mn concentrations and Φ_PSII_/ETR) were created in OriginPro^65^ and improved using iterative least squares *via* the implemented Levenberg-Marquardt algorithm (Tab. S7). Model fit significance was evaluated using p-values for each parameter. Fits were considered converged at a χ² tolerance of 1 × 10^− 9^. Fit quality was assessed using the coefficient of determination (r^2^) with statistical significance determined by rejecting the null hypothesis, that the fitting function is not significantly better than the function y = 0, at *p* < 0.05.

## Results

The data repository includes all raw data generated in this study.

### Tephra characterisation

#### Particle size distribution

All tephra particles used in the experiments are fine volcanic ash < 250 μm in diameter^66,67^. However, the particle size distribution of the different samples significantly varied (Tab. 2). T1 and T2 exhibited the largest median particle sizes, measuring 233.67 ± 24.41 μm and 142.10 ± 2.86 μm, respectively. In contrast, T3 and T4 had the smallest median particle sizes, at 34.07 ± 1.22 μm, and 59.42 ± 0.71 μm, respectively (Tab. 2). Sorting was influenced by the median particle size, with T1 and T2 displaying greater sorting coefficients (184.14 ± 26.62 μm, and 125.50 ± 6.00 μm) compared to T3 and T4, which both have sorting values of ≈ 65 μm (Tab. 2). As expected for crushed particles, these values are characteristic of well sorted tephra deposits^68^.

**Table 2:** Tephra particle size distributions. Data are presented as mean ± standard deviation (SD) (*n* = 3).

Tephra sample	Median particle size [µm]	Sorting σ* [µm]	Inter-aliquot variability [%] *n* = 3
T1	233.67 ± 24.41	184.14 ± 26.62	10.17
T2	142.10 ± 2.86	125.50 ± 6.00	3.67
T3	34.07 ± 1.22	68.03 ± 4.66	5.04
T4	59.42 ± 0.71	62.30 ± 0.72	1.06

* σ = $\:\frac{{D}_{84}-{D}_{16}}{2}$, with Dx = xth percentile of the cumulative particle size distribution.

#### XRD

**Fig. 3:**
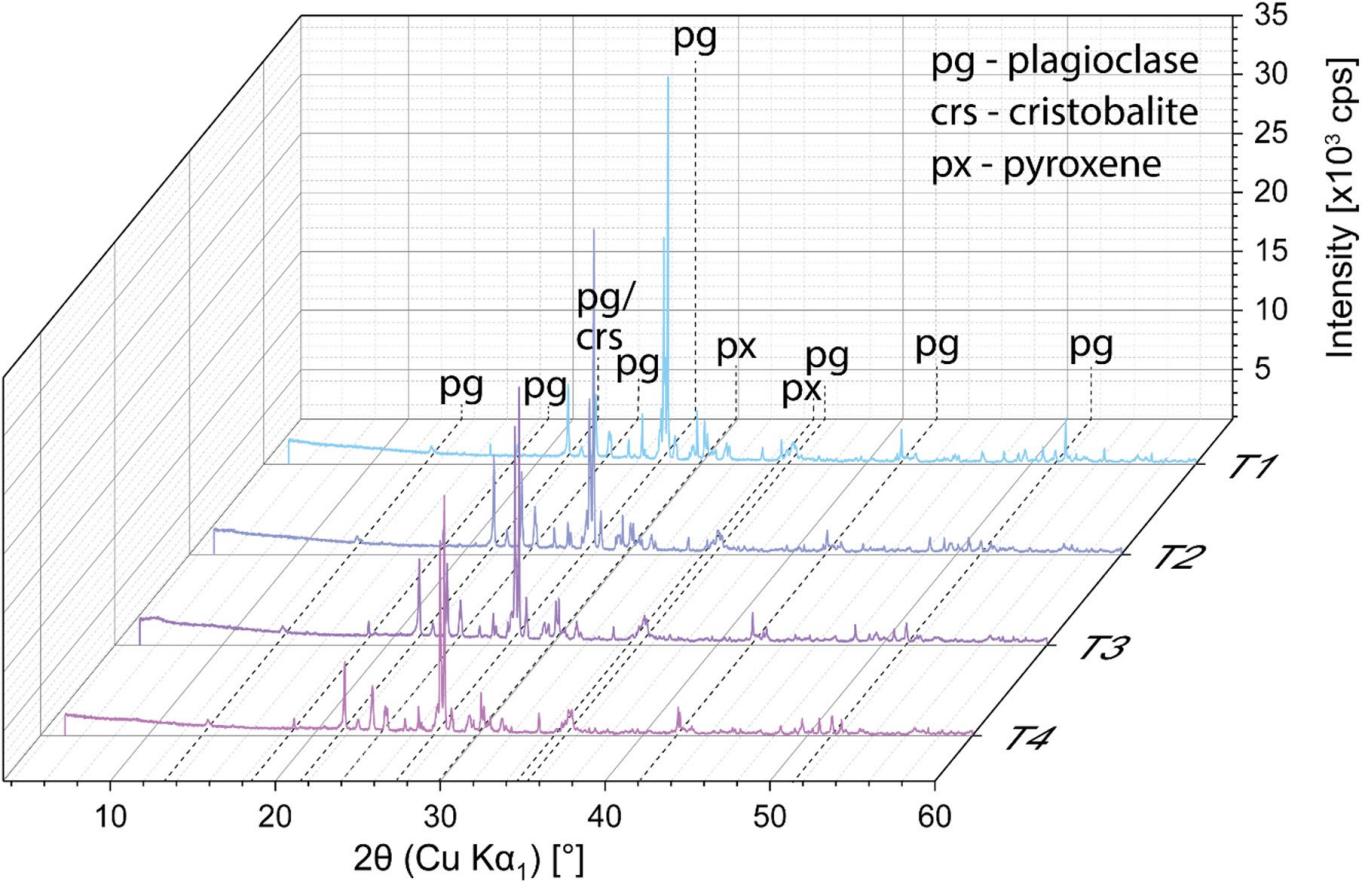
XRD diffractograms of ground tephra samples T1, T2, T3 and T4. The colour scale is used to distinguish between samples.

The XRD patterns of all tephra samples (T1, T2, T3 and T4) revealed the presence of a dominant Ca-rich plagioclase feldspar phase in the mineral fraction (Fig. 3). A semi-quantitative phase determination, based on the measured peak intensity over the reference intensity using the Diffrac.Eva software showed 80–90 wt% of feldspar in the mineral fraction of all samples, with pyroxene (5–15 wt%) and cristobalite (< 5 wt%) present as the second and third most abundant phases, respectively. The diffractograms of all tephra samples are dominated by the plagioclase peaks at ≈ 28° (2θ) (Fig. 3).

#### Bulk geochemical composition of tephra

The bulk rock chemistry classifies all four tephra samples as basaltic andesite with a total alkali content (Na_2_O and K_2_O) of ≈ 3.5 to 4.2 wt% and silica contents ranging from 53.0 to 54.5 wt% (Fig. S2 & Tab. S2). Major and minor elemental compositions of the bulk are indistinguishable between the tephra samples, as reflected, for example in the comparable MnO contents in T1 (0.18 wt%), T2 (0.21 wt%), T3 (0.17 wt%), and T4 (0.17 wt%).

#### Volcanic glass quantification via SEM-EDS

Mineral and glass fractions were determined for each tephra sample using particle characterisation and counting (Tab. S3). Volcanic glass constituted approximately 30% of the tephra samples T1 (34.6%, 95% CI: 21.7 to 47.6%), T2 (27.7%, 95% CI: 16.8 to 38.6%), and T3 (29.8%, 95% CI: 18.0 to 41.7%). Only the pristine ash sample T4 exhibited a comparatively lower glass content of 18.0% (95% CI: 7.4 to 28.7%), although this value still overlapped with the confidence intervals of the other samples.

#### Geochemical composition of volcanic glasses

After data cleaning and application of the multivariant outlier test (described in Sect. 2.1.5 & 2.5), the major and minor element compositions of volcanic glass from the tephra samples are reported as medians in Tab. S4. The data repository contains the corresponding cleaned dataset with outliers removed. With SiO_2_ contents ranging from ≈ 63 to 71 wt%, and alkali contents (Na_2_O and K_2_O) of ≈ 6 wt%, the volcanic glass from all tephra samples classifies predominantly as subalkaline dacite (Fig. S2). The chemical compositions of volcanic glass are indistinguishable between the tephra samples, as reflected, for example, by comparable MnO contents in T1 (0.18 ± 0.03 (*SD*) wt%), T2 (0.17 ± 0.05 (*SD*) wt%), T3 (0.18 ± 0.04 (*SD*) wt%), and T4 (0.17 ± 0.04 (*SD*) wt%).

### Seawater Mn concentrations

**Fig. 4:**
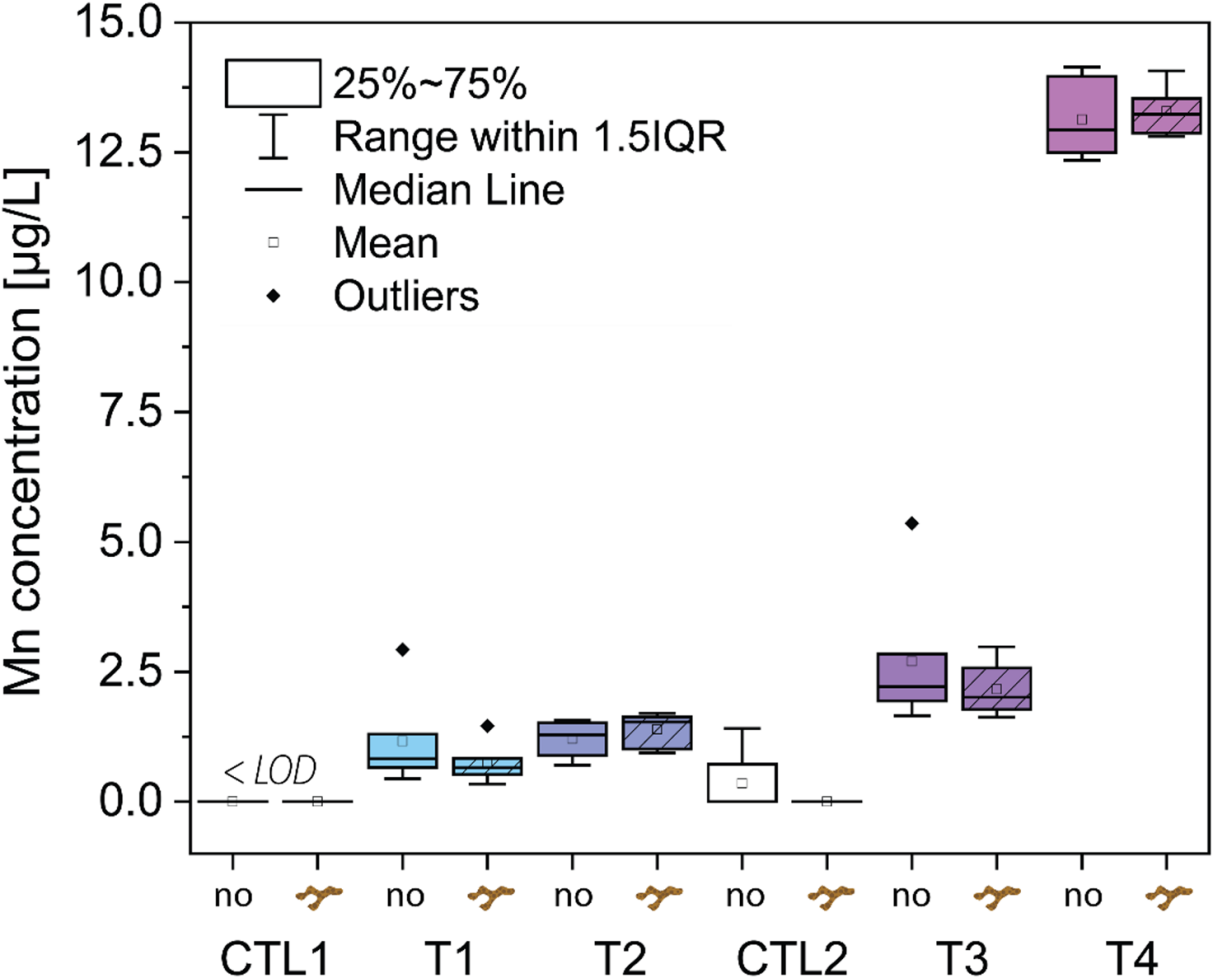
Seawater Mn concentrations [µg L^− 1^] of control (Ctl1 & Ctl2) and tephra-exposed conditions (T1-T4). Control represents seawater blanks. Each condition contains two boxplots, one representing the measured Mn values in beakers without and the other with three nubbins of *S. pistillata* (plain and striped box, respectively). In total, each box and whisker plot contain six observations, corresponding to the weekly measurements for duplicate sets of beakers. The colour scale is used to distinguish between samples.

All tephra samples leached Mn, which resulted in increased Mn concentrations across all experimental conditions, whereas seawater blanks remained below LOD (< 0.2 µg L^− 1^) (Tab. S6; Fig. 4). T4 (pristine ash) leached the most Mn (13.13 ± 0.69 (*SD*) µg L^− 1^), followed by T3 (2.70 ± 1.24 (*SD*) µg L^− 1^), T2 (1.21 ± 0.33 (*SD*) µg L^− 1^) and T1 (1.16 ± 0.83 (*SD*) µg L^− 1^) in non-coral seawater, which corresponded to a 69-, 14-, 5- and 4-fold increase in Mn, assuming the Mn content in the control condition equals the LOD (Tab. S6). When comparing the effect of coral nubbins on the Mn concentration in the beaker under a given condition, no significant difference was observed compared to the beaker without coral (Welch, *p* > 0.2 across all conditions). Hence, the presence of corals did not change the measured Mn concentration (Tab. S6).

### Light measurements

**Fig. 5:**
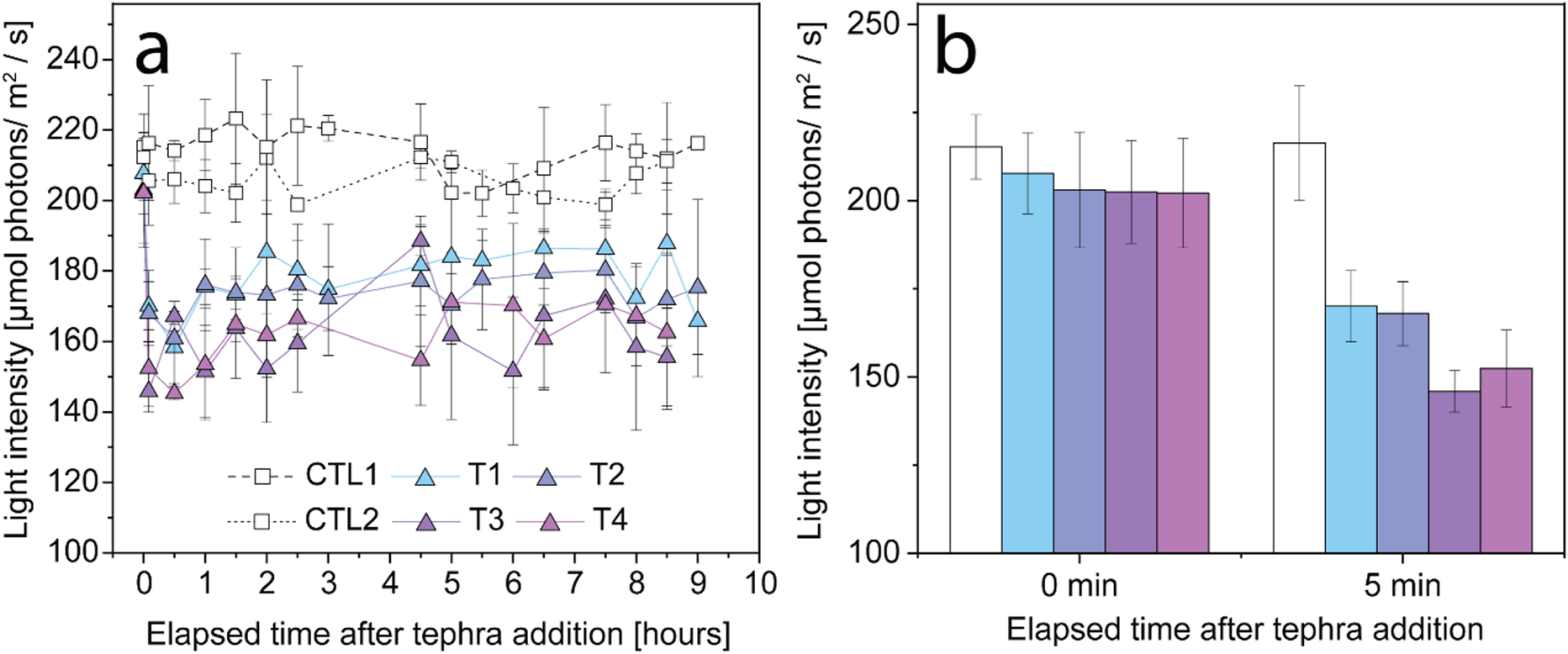
Changes in light intensity following tephra addition. **(a)** Daily monitored light intensity in each non-coral containing beaker, measured over 9 h at various time steps over the three weeks experiment. The sample number varies depending on the respective time step. **(b)** Measured irradiance, before and 5 min after tephra addition (*n* = 18), highlighting the onset of turbidity. Data are presented as mean ± SD. The colour scale is used to distinguish between samples.

The addition of tephra to the beakers led to measurable changes in PAR. The instantaneous increase in turbidity (and reduction in measured irradiance) was maintained throughout the day (Fig. 5). The drop in light intensity (between 35 and 56 µmol photons m^− 2^ s^− 1^) was immediate and similar under all the experimental conditions (Welch, *p* < 0.0001). Of all tephra samples, the in situ tephra (T3 and T4) decreased PAR the most (49.70 and 56.51 µmol photons m^− 2^ s^− 1^, respectively). The measured irradiance in the control beaker did not change over the day (Welch, *p* = 0.81).

### Physiological measurements

**Fig. 6:**
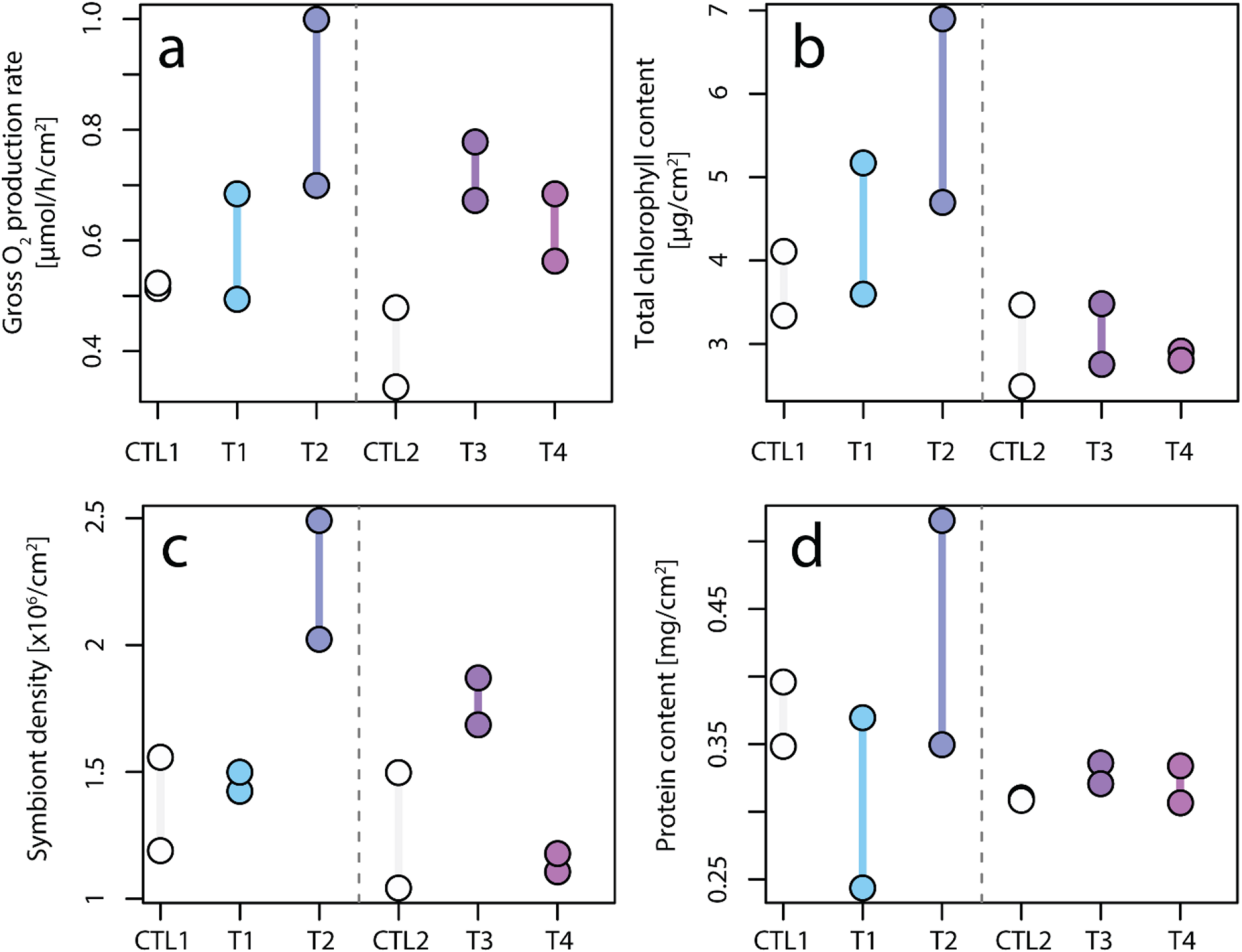
Physiology of *S. pistillata* reared in control conditions (in white) and after 3 weeks of daily exposure to various tephra samples (1 g L^− 1^). Selected physiological parameters are expressed as (**a**) gross oxygen production rate, (**b**) total chlorophyll *a* + *c*_*2*_ content, (**c**) dinoflagellate density as the number of symbionts, and (**d**) total protein holobiont content, all normalized per skeletal surface area. Individual measurements are plotted as points (*n* = 2 per condition). The colour scale is used to distinguish between samples.

The daily addition of the four different tephra samples appears to have resulted in elevated gross oxygen production (Fig. 6a) in all four conditions. Compared to control experiments, oxygen production per surface area after 3 weeks led to a 1.7-fold (T1), 1.8-fold (T4), 2.5-fold (T2) and 2.6-fold (T3) increase in P_n_ (Fig. S3b) and a 1.1-fold (T1), 1.5-fold (T4), 1.6-fold (T2) and 1.8-fold (T3) increase P_gross_ (Fig. 6a; Fig. S3a). Conversely, no obvious change in respiration was observed following the addition of tephra (Fig. S3d&e). For the remobilised tephra samples (T1 & T2), an increase in total chlorophyll content (a + c_2_) was apparent compared to control conditions, on the contrary to the in situ tephra (Fig. 6b). This change was driven by changes in chlorophyll *a* (Fig. S4b&c), although condition T1 (marine deposit) resulted in lower chlorophyll *c*_*2*_ content per surface area (Fig. S4d). Symbiont density (per area) showed no change compared to control conditions for T1 and T4, but increased by 1.6- and 1.4-times for T2 and T3 compared to the reference control (Fig. 6c). In general, protein content remained similar across all conditions (around 0.35 mg cm^− 2^; Fig. 6d), although large intra-condition variations for T1 and T2 makes interpretation difficult.

#### Chlorophyll fluorescence measurements

**Fig. 7:**
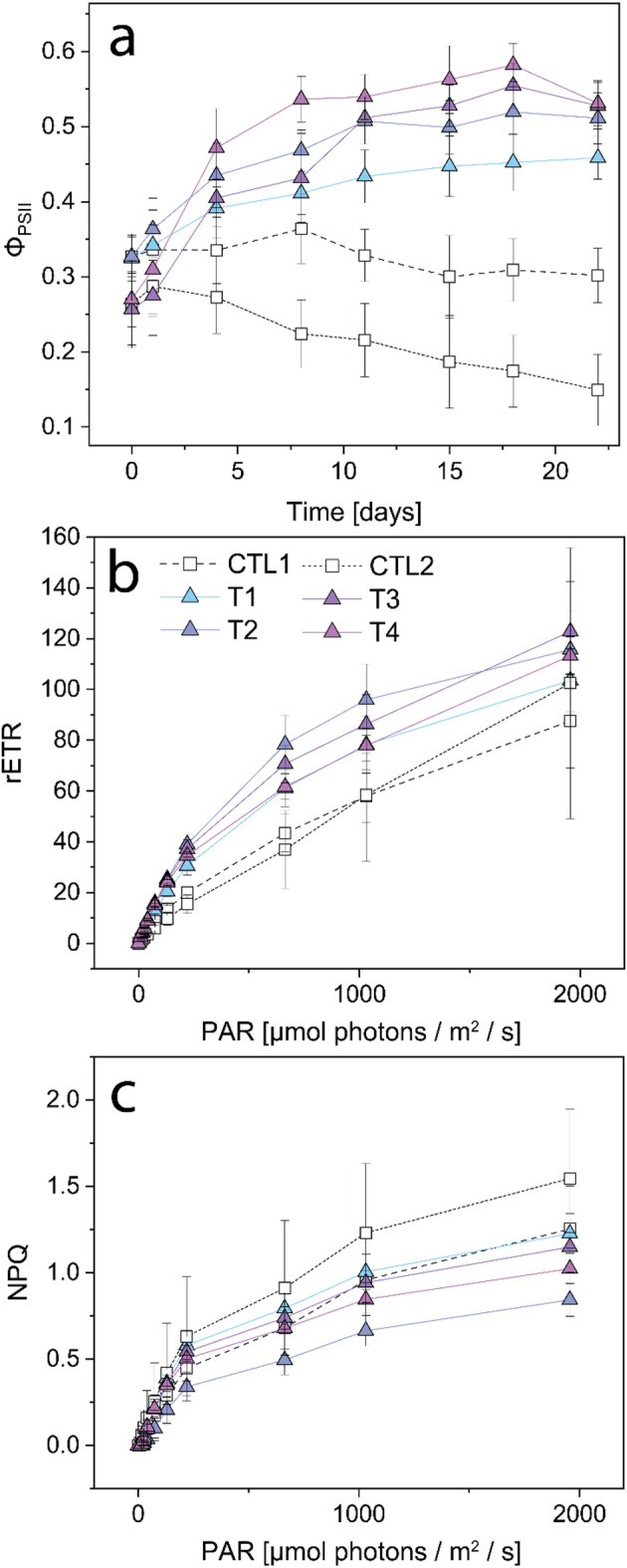
Chlorophyll fluorescence-derived photophysiological parameters of *S. pistillata* reared in control conditions (in white) and after 3 weeks of daily exposure to various tephra samples (1 g L^− 1^). (**a**) The effective quantum yield of PSII (Φ_PSII_) of light-adapted nubbins throughout the experiment (*n* = 6 per condition). The light response of dark-adapted *S. pistillata* to rapid light curves (RLC) was measured as (**b**) the relative electron transport rate (rETR), and (**c**) the non-photochemical quenching (NPQ) (*n* = 6 per condition). Data are presented as mean ± SD based on observations from six nubbins. The colour scale is used to distinguish between samples.

Fluorescence-derived photochemical parameters of PSII were significantly impacted by the addition of tephra. While all nubbins exhibited average effective Φ_PSII_ values between 0.26 ± 0.05 (Ctl2, T3 & T4) and 0.33 ± 0.03 (Ctl1, T1 & T2) at the start of the experiment (Fig. 7a), the exposure to all four tephra samples significantly increased Φ_PSII_ after four days compared to the controls (ANOVA, *p* < 0.001, across all conditions). This increase approached a plateau between 0.46 ± 0.03 and 0.58 ± 0.03 during the experiment (Fig. 7a). Nubbins from T3 and T4 started at significantly lower Φ_PSII_ than T1 and T2 (ANOVA, *p* < 0.002 at day 0), but increased to similar Φ_PSII_ after four days. From all tephra samples, T1 increased the least after three weeks (1.4 times), while T3 and T4 experienced the largest improvements in Φ_PSII,_ with values 2 times higher than at the start of the experiment.

The RLC-derived parameters (ETR and NPQ; Fig. 7b & c) were significantly affected after three weeks of tephra exposure. The ETR was significantly higher for all tephra-exposed nubbins compared to the controls (ANOVA, *p* < 0.0001). The significant increases in ETR for tephra conditions remained over a vast range of irradiances, but diminished at high light conditions (> 1032 µmol photons m^− 2^ s^− 1^), where control and experimental conditions displayed similar values (Fig. 7b). ETR levels in Ctl2 nubbins were significantly lower than in Ctl1 nubbins at low light conditions, but after 130 µmol photons m^− 2^ s^− 1^, these differences vanished. NPQ levels did generally not change in response to the prolonged tephra exposure (Fig. 7c), except for T4 compared to Ctl2 nubbins at high light conditions (PAR = 1032 µmol photons m^− 2^ s^− 1^, ANOVA, *p* < 0.05; PAR = 1956 µmol photons m^− 2^ s^− 1^, ANOVA, *p* < 0.006). Clt2 nubbins displayed the highest NPQ levels of all experimental conditions.

#### Skeletal growth rate

**Fig. 8:**
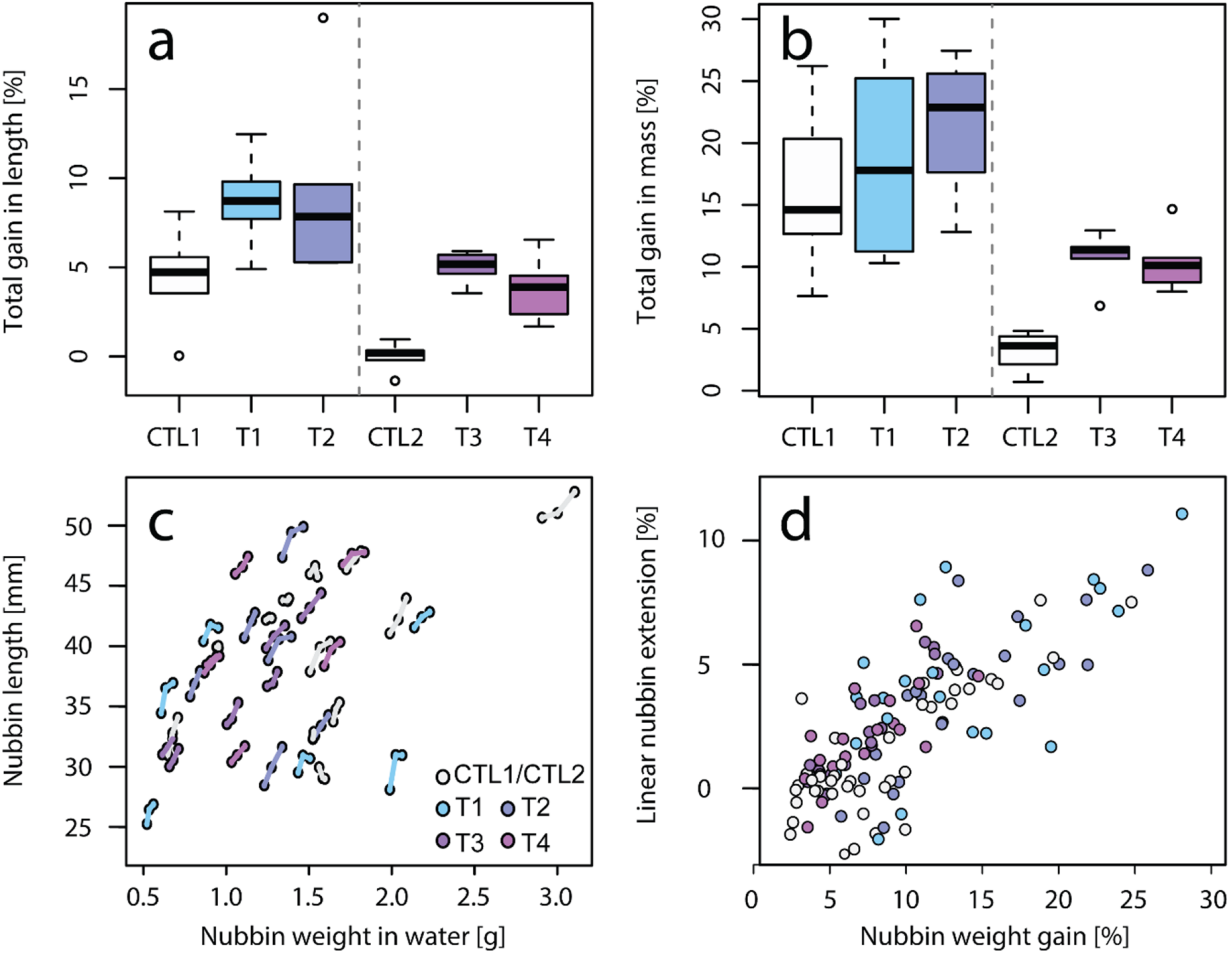
Skeletal growth rate of *S. pistillata* reared in control conditions (in white) and during 3 weeks of daily exposure to various tephra samples (1 g L^− 1^). The growth rate is expressed as the average (**a**) linear extension rate and (**b**) skeletal mass gain of nubbins after three weeks of exposure (*n* = 6 per condition). Results of the weekly skeletal growth of individual nubbins are presented as (**c**) the relationship between nubbin length and their weight, and (**d**) the extension and weight gain relative to their initial values. The colour scale is used to distinguish between samples.

Skeletal growth, measured either as extension or mass gained, tended to be higher in tephra-exposed nubbins compared to the respective control nubbins but was only statistically significant for T3 vs. Ctl2 (Welch, *p* < 0.04). Although not significantly different for T1 and T2 compared to Ctl1, the respective rate of skeletal extension was 95.65% and 105.11% higher (Fig. 8a), and the average rate of skeletal mass gain was 17.01% and 34.56% higher (Fig. 8b). T3 and T4 nubbins showed higher extension rates relative to Ctl2 (Fig. 8a), but since Ctl2 extension rates were close to zero (0.02 ± 0.71%), these results should be interpreted with caution. Over a three-week duration, both skeletal extension and skeletal mass gain (3.21 ± 1.40%) were lower in Ctl2, than in Ctl1 nubbins (4.46 ± 2.42%, and 16.01 ± 5.96%, respectively). A similar effect was seen between the tephra-exposed corals, where T3 & T4 showed lower skeletal growth compared to T1 & T2 (extension gain: 4.42 ± 1.38% vs. 8.94 ± 3.68%; weight gain: 10.59 ± 2.02% vs. 20.14 ± 6.65%). Linear extension and skeletal mass gain appeared to be positively correlated, with no clear distinction between individual conditions (Fig. 8c & d).

### Correlation of photosphysiological parameters with seawater Mn concentration

**Fig. 9:**
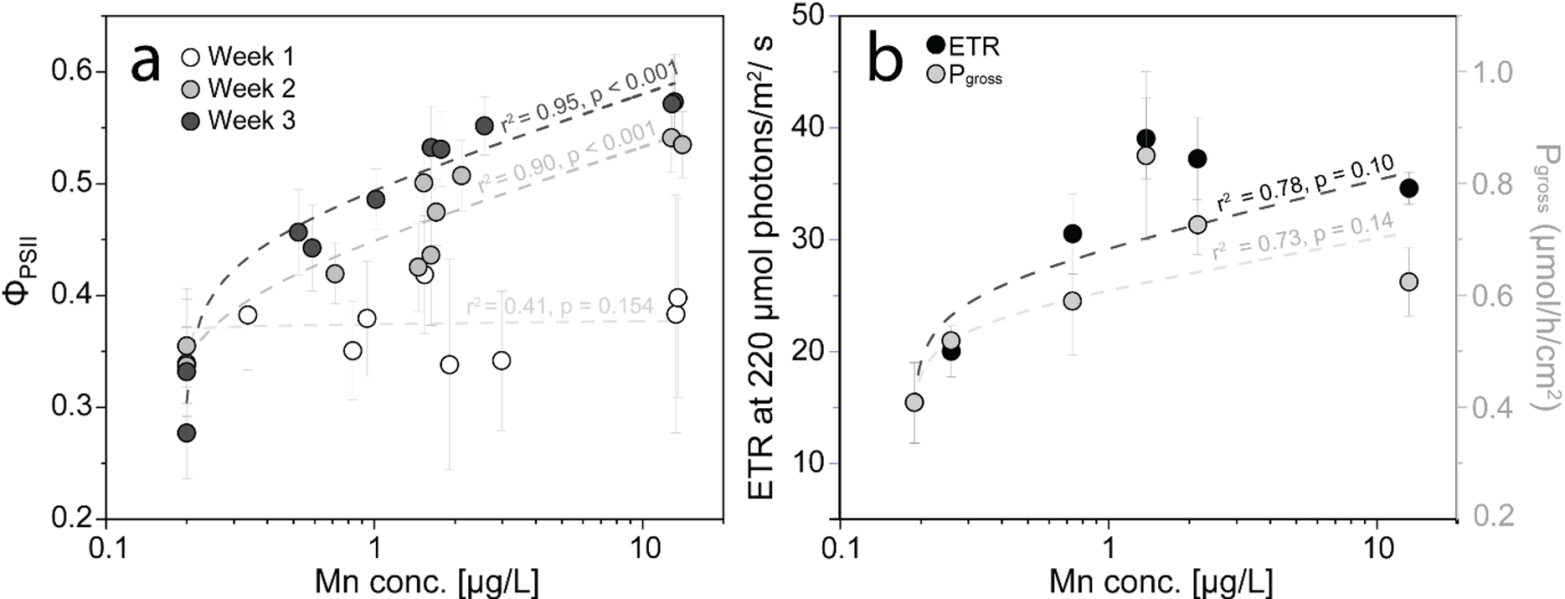
Relationship between seawater Mn concentrations [µg L^− 1^] and the measured photophysiological response of *S. pistillata* nubbins in control and tephra-exposed conditions. Photosynthetic parameters include **(a)** Φ_PSII_ over the three-week experiment (*n* = 3 per sample, totaling *n* = 18 per condition), and **(b)** RLC-derived ETR (*n* = 6 per condition) and P_gross_ normalized per surface area (*n* = 2 per condition), measured at the end of the experiment. Data are presented as mean ± SD. The error of the seawater Mn concentration was calculated from the relative standard deviation of two duplicate measurements. Lines were fitted to each of the datasets using a logarithmic model (y = a–b*ln (x + c)), with the fits summarised in Tab. S7.

Some chlorophyll fluorescence-derived parameters such as Φ_PSII_ or ETR and P_gross_ showed strong logarithmic correlations (r^2^ = 0.41–0.95) with measured Mn concentrations in the coral-equipped beakers (Tab. S7), especially after prolonged exposure (Fig. 9). For the case of Φ_PSII_, significant correlations were observed after two weeks of constant tephra exposure with improving correlations week by week (Fig. 9a). In the case of ETR at 220 µmol photons m^− 2^ s^− 1^ (Fig. 9b), which resembles the closest light intensity to which corals were exposed, correlation was not significant (r^2^ = 0.78, *p* = 0.10). Similarly, no significant correlation was detected for P_gross_ normalized to surface area (r^2^ = 0.73, *p* = 0.14). This behavior was observed independently of the tephra sample. However, the logarithmic dependency of photosynthetic parameters on Mn concentration was not observed for NPQ.

## Discussion

### Leaching of Mn from tephra drives coral photophysiology

Although the pristine ash from the La Soufrière eruption leaches a wide range of essential trace metals^69^, Mn was the most extensively released and showed the highest bioaccumulation in coral tissue^70^. A recent study by Moreira, Júnior^32^ highlights the positive effect of Mn alone on photosynthesis of *S. pistillata*, supporting a likely link between the water-soluble Mn leached from tephra and the observed enhancement in photosynthesis. Leaching of Mn led to optimized photosynthetic efficiency of PSII in tephra-exposed nubbins of *S. pistillata* after three weeks, irrespective of tephra sample and thus irrespective of the amount of Mn leached (Fig. 7). This is evident in the unanimously higher Φ_PSII_ values after three days of exposure (Fig. 7a), ETR (Fig. 7b), P_gross_ (Fig. 6a) and P_n_ (Fig. S3b&c) compared to the control conditions. Bioavailable Mn is a key trace element for coral symbionts^33^, and although different tephra samples leach varying amounts of Mn, the positive photophysiological response of the holobiont remained similar. This suggests that a small increase in the total dissolved Mn concentration (1–3 µg L^− 1^) is enough to boost coral health, irrespective of tephra remobilisation, and is supported by similar photo-physiological responses at a lower tephra loading^23^. This is seen in the apparent logarithmic dependency of photosynthetic parameters on seawater Mn content after three weeks exposure (Fig. 9). While a rapid increase in Φ_PSII_ and rETR of PSII occurs at Mn concentrations of less than 3 µg L^− 1^, this increase stagnates at Mn concentrations in seawater between 5 and 15 µg L^− 1^. The exchange of Mn between coral nubbins and seawater could not be quantified within the daily incubations, as Mn concentrations remained the same between coral and non-coral beakers (Fig. 4). This can be due to the daily replenishment of seawater with new tephra, or the fact that Mn uptake rate by the coral nubbins was within measurement error or extremely low (below 1 µg L^− 1^). The mutualistic endosymbiosis of Symbiodiniaceae dinoflagellates and their reef-building coral hosts is specialized for thriving in oligotrophic to mesotrophic waters, spanning from low to high nutrient regimes^71^. We propose that even slight increases in Mn content, supplied by tephra exposure, can alleviate enzyme-specific metal limitations and enhance photosynthetic performance over both short-term (days) and long-term (weeks) exposure durations. Under such conditions, the micronutrient-limitation of coral photosynthesis may instead become constrained by macronutrient (i.e., phosphate and nitrate) availability. Corals may increase their nitrate uptake from seawater, or tephra itself may release nitrate together with metals, but this has not been investigated in this study.

### Enhanced coral resilience under turbidity and sedimentation stress

The leached Mn concentrations (between 1 and 13 µg L^− 1^, depending on tephra sample) were far below chronic toxic Mn levels reported for the branching coral *Acropora millepora*, for which no toxic effect (tissue sloughing) was observed after a two-week exposure to 1090 µg Mn L^− 1^^35^. In addition to potential metal toxicity after exceeding species-specific thresholds, nubbins exposed to tephra were subjected to various other stressors including: (i) persistent turbidity following daily tephra addition (1 g L^− 1^), expressed as a constant decrease in PAR equal to 50 µmol photons m^− 2^ s^− 1^ (equalling 25% reduction in ambient PAR; Fig. 5); (ii) sedimentation stress; and (iii) mechanical abrasion through contact with sharp glassy particles. However, tephra-exposed nubbins of *S. pistillata* did not show signs of elevated stress but rather reduced stress compared to the corresponding control corals. In the case of control nubbins, photoautotrophy could not satisfy maximal daily energy requirements even during optimal light conditions, which led to a persistent decrease in photosynthetic yields. However, once exposed to either tephra sample, nubbins rapidly photoacclimatized and maintained high levels of photo efficiency even at high turbidity levels (Figs. 5 and 7). This improved photophysiology (Fig. 7), resulting from volcanic tephra leaching, led to increased rates of photosynthesis, as indicated by increased O_2_ production^23^ (Fig. 6a), and was associated with increased coral growth (Fig. 8), reflected in greater extension rates in T3 nubbins compared to Ctl2 nubbins (ANOVA, *p* < 0.04; Fig. 8a).

Sedimentation and turbidity are known environmental stressors that can lead to reduced photosynthetic efficiency in symbiotic corals [reviewed in^10,72^], with short-term and prolonged sediment loading negatively impacting coral growth, photophysiology, dinoflagellate density and chlorophyll content^7,20,73^. Tephra deposition and remobilisation into coastal water systems affect turbidity and total dissolved solids contents in the water body over months and years after the eruption^74^, with the largest changes occurring shortly (days) after the eruption^75^. Coral reefs surrounding volcanic islands might therefore expect prolonged high sedimentation loads and light reduction associated with syn-eruption deposition and post-eruption remobilisation of volcanic tephra.

Turbidity is influenced by both the sediment load, and their particle size distribution. T1 (233.67 ± 24.41 μm) and T2 (142.10 ± 2.86 μm) had larger median particle sizes than T3 (34.07 ± 1.22 μm) and T4 (59.42 ± 0.71 μm), which led to smaller decreases in light intensity following tephra addition (Fig. 5; T2 [−35.06 PAR]; T1 [−37.64 PAR]; T4 [−49.69 PAR]; T3 [−56.51 PAR]). This particle size effect on turbidity is in agreement with observations by Meral^76^. The addition of 1 g L^− 1^ tephra exceeds the limit of most laboratory sedimentation loading studies, which cap their highest sediment rates at 100–200 mg L^− 1^ [e.g. ^20,77^]. Even at a turbidity level of 1 g L^− 1^, we did not observe decreases in the symbiont density, total chlorophyll content and/or photosynthetic yields (Figs. 6b and c and 7a). In contrast, we observed significantly higher Φ_PSII_ and rETR at irradiances < 1000 µmol photons m^− 2^ s^− 1^ in tephra-supplied corals compared to their controls (Fig. 7). This corresponded to an increase in P_n_ and P_gross_ at ambient PAR (Fig. 6a & S3), highlighting the positive relationship between yield and oxygen production at low light intensities (< 200 PAR)^56,78^. Coral bleaching was also not observed in any experimental condition. In contrast, exposure to tephra samples T2 and T3 resulted in increased symbiont densities by 1.5- to 2-times compared to the control (Fig. 6c). This improved efficiency of the photosynthetic apparatus at high turbidity highlights the adaptability of *S. pistillata* to a wide range of light intensities^47^. Adaptability for other stressors, such as sediment loading thresholds and the sensitivity to it, is coral-specific^72^. For example, branching corals such as *S. pistillata* are generally more resilient than thin-tissued and flat coral morphotypes^79^. Potential coral damage does not only depend on sedimentation loading (amount and duration) but also strongly depends on the sediment type. Tissue degradation was shown to increase with increasing organic content in the sediment^80^ and bacterial activity. Microbiological analyses conducted on pristine St. Vincent ash from 2021 did not detect bacteria^81^, which are present in other geogenic particles such as desert dust^81^. This biological component may play a role in the cellular response of corals to the sedimentation stress. It needs to be tested how the microbial colonization of the different tephra samples varies in reference to pristine volcanic ash (T4).

### Mn budgets from pristine and weathered tephra

The daily addition of various tephra samples (1 g L^− 1^) from the island of St. Vincent into coral-containing beakers led to a measurable (4- to 60-times) increase in seawater Mn concentrations (Fig. 4). We observe that the amount of Mn leached was largely governed by the origin of the tephra (Fig. 4). Pristine volcanic ash supplied the largest amount of Mn (13.13 ± 0.69 (*SD*) µg L^− 1^) to the seawater, while the altered tephra samples leached between 1 and 3 µg L^− 1^ (Tab. S6). This result may appear counterintuitive, given that the bulk mineralogical and chemical composition of all four tephra samples was identical as indicated by XRD (Fig. 3) and XRF analysis (Tab. S2). The estimated volcanic glass contents were also comparable ranging from 18.0% to 34.6% (Tab. S3), with T4 displaying the lowest glass fraction, estimated at 18.0% (95% CI: 7.4 to 28.7%). However, overlapping confidence intervals among these samples suggest no statistical differences in glass contents. Furthermore, none of the XRD diffractograms exhibited the characteristic broad hump at ≈ 25° (2θ), typically associated with a dominating volcanic glass phase. Previous work by Rowe, Ellis^82^ demonstrated that such an amorphous silica peak is observable even at glass contents of ≈ 40 wt%. The absence of this feature in our samples implies that the actual glass contents may lie toward the lower bounds of the estimated confidence intervals (ranging from 7.4% in T4 to 21.7% in T1). EPMA analysis on the volcanic glass particles revealed no difference in the Mn contents of the four tephra samples (≈ 0.18 wt% MnO; Tab. S4), confirming the results obtained by Weber, Blundy^42^. Consequently, we were unable to distinguish the Mn leaching source between individual tephra samples based on their mineralogy (Fig. 3), glass content (Tab. S3), bulk chemistry (Tab. S2), and glass compositions (Tab. S4), which are all comparable or nearly identical between samples.

To understand differences between the tephra samples, we must understand how their depositional history could influence the potential source of Mn. Each sample experienced varying degrees of weathering and contact times with water. T1 and T2 were remobilised tephra transported and deposited through a river system, with a key distinction: T1 was deposited in a marine delta, while T2 was sampled from a river mouth behind the coastline (Fig. 1). The PDC deposit (T3) near the summit crater originated from the 2021 eruption and has remained largely unchanged since deposition. In contrast, the pristine ash (T4) sampled in Georgetown is largely unaltered, even though it likely contains non-juvenile material originating from the fragmentation of lava domes^41^. Unlike T1, T2 and T3, it has not been exposed to rain or seawater after deposition. The highest leaching associated with pristine tephra is in agreement with volcanic ash leaching studies^43,83,84^, which highlight the fertility of pristine ash, due to the quick dissolution of surface-adhering metal salt encrustations that form following in-plume reactions between hot volcanic gases, condensing acid droplets and tephra particles^85,86^.

Another factor influencing the leaching of Mn could be variations in particle size between samples. All tephra samples are composed of fine volcanic ash particles < 250 μm, but exhibit differences in particle size and distribution that can affect the leaching potential, as smaller tephra particles tend to have a higher surface area to mass ratios than larger particles^87^. This results in a higher adsorption capability of leachate species, and allows for more seawater-mineral/glass interaction, leading to increased water-soluble metal release^43^. Among the altered tephra samples, T3, with the smallest median particle size (34.07 ± 1.22 μm), released ≈ 2.2 times more Mn than T1 and T2 (233.67 ± 24.41 μm and 142.10 ± 2.86 μm, respectively). This points at a particle size dependence on the leaching rate as shown for other mineral aerosol particles^88^. However, particle size is not the primary factor influencing leaching, as freshly erupted and unaltered volcanic ash (T4), although larger in median size (59.42 ± 0.71 μm) compared to T3, leached 4.9-times more Mn than T3.

We propose that the Mn leaching potential of tephra is governed by a combined effect of particle surface chemistry and size. The latter contributes through increased surface area, which is further enhanced by ash vesicularity and surface roughness^87^. Explosive volcanic eruptions produce vast quantities of pristine silicate tephra, with particles exhibiting substantial differences in their surface chemistry due to nanosilicate melt heterogeneities^89^. While X-ray photoelectron spectroscopy (XPS) could provide important insights into the presence of Mn-bearing surface phases, the low concentrations involved often fall below detection limits as seen in Hornby, Ayris^89^. Thus, we emphasize that the unique surface chemistry of volcanic ash, coupled with particle-size effects, controls the initially high Mn solubility of pristine tephra in seawater, which decreases substantially with prolonged exposure and after particle settling. The exposure time to river and seawater could also explain why the submarine-deposited sample T1 and the river delta-deposited sample T2 leached the lowest Mn concentrations (1.16 ± 0.83 (*SD*) µg L^− 1^ and 1.21 ± 0.33 (*SD*) µg L^− 1^, respectively). Given that coral reefs are more likely to be exposed to remobilised and resuspended rather than pristine tephra, further research will be necessary to quantify the long-term physiological impacts of such exposure both at organismal and reef levels.

### Experimental limitations

This study aimed for a mechanistic understanding of how exposure to tephra of different depositional settings and water-contact histories affect the physiology of scleractinian corals. The experimental design (i.e., 12 beaker incubations, a single coral species, and a tephra dosage of 1 g L^− 1^) was not intended to replicate natural tephra runoff concentrations, which remain understudied. Instead, the chosen concentration follows previous ash-leachate studies to highlight acute physical and chemical effects of tephra addition. Daily additions (five times per week) approximate repeated short-term inputs, which might occur for corals living few kilometres away from the eruption event, or by corals living close to the eruption and receiving remobilised tephra years after the eruption.

Certain methodological choices limit interpretation: (i) The daily transfer of nubbins to a regeneration tank does not reflect reef conditions but may mimic dilution in open-ocean settings, especially when sediment supply is not continuous. (ii) Tephra samples were crushed but not homogenised to a uniform particle size, potentially influencing leaching rates. Additionally, the crushed tephra are not identical to natural tephra samples, for which the grainsize distribution is controlled by different physical processes, including the type of volcanic hazard (i.e., better sorted ash fall deposits vs. poorly sorted PDC deposits). (iii) Water chemistry (pH, salinity, and macronutrients such as phosphate and nitrate) was not monitored, restricting insights into drivers of skeletal growth responses. (iv) The experimental duration (three weeks) contrasts with real reef exposure, which can persist for years and decades^4^. (v) Furthermore, growth rate variations between control groups (Ctl1 and Ctl2) suggest possible genetic differences among nubbins or different stress levels. This variability could have been potentially avoided by using fragments from a single mother colony.

## Conclusions

In controlled laboratory settings, coral nubbins of *S. pistillata* were exposed for three weeks to different tephra samples collected during and after the recent eruption of the La Soufrière volcano on St. Vincent. The tephra was sieved and crushed to comparable particle sizes, and while the bulk mineralogical and chemical composition, volcanic glass content and its composition were broadly similar across the different tephra, variations arose due to differences in the median particle size, the depositional settings and water-contact history. Seawater Mn concentrations were measured weekly in beakers, both with and without coral nubbins. Tephra released significant but non-toxic amounts of Mn (1–13 µg L^− 1^, depending on the type), resulting in consistent increases in the light-harvesting efficiency of Symbiodiniaceae within the coral animal tissue (evident in the increased P_n_, P_gross_, and rETR). This enhancement in photosynthetic activity persisted throughout the experiment (evident in high Φ_PSII_-levels), leading to measurable changes in coral growth, while other physiological parameters, such as protein and chlorophyll content, remained largely unchanged. Despite causing sedimentation and increased turbidity, tephra exposure optimized the light-harvesting ability of Symbiodiniaceae in *S. pistillata*. In tephra-exposed *S. pistillata* the increased availability of photosynthates from photosynthetic upregulation may enhance resilience against environmental stresses imposed by tephra addition. Our data highlights the critical role of tephra-supplied Mn to coral organisms, showing that even weathered and remobilised tephra deposits can leach biologically relevant concentrations. Given the potential for enhanced post-eruptive sediment transport, terrestrial runoff and remobilisation of tephra may persist over timescales of years to decades. Further research is needed to understand the long-term input of tephra-sourced Mn and its role in coastal Mn cycling and to validate these findings in natural reef environments.

## Supplementary Information

Below is the link to the electronic supplementary material.

Supplementary Material 1

## Data Availability

The original data that support the findings of this study are available in Mendeley Data with the identifier doi:10.17632/np9bhzfp56.3.
