# Supplementary material for: Tephra-mediated manganese cycling shapes coral responses to coastal sedimentation
Source: Sci Rep. 2026 Feb 4;16:7216. doi: 10.1038/s41598-026-38388-9 (PMC12923829; doi:10.1038/s41598-026-38388-9)
Supplement: Supplementary file 1 — Supplementary Material 1 [file 41598_2026_38388_MOESM1_ESM.docx]

**Supplementary Material for
“Tephra-mediated manganese cycling shapes coral responses to coastal sedimentation*” (Förster et al., 2025)***


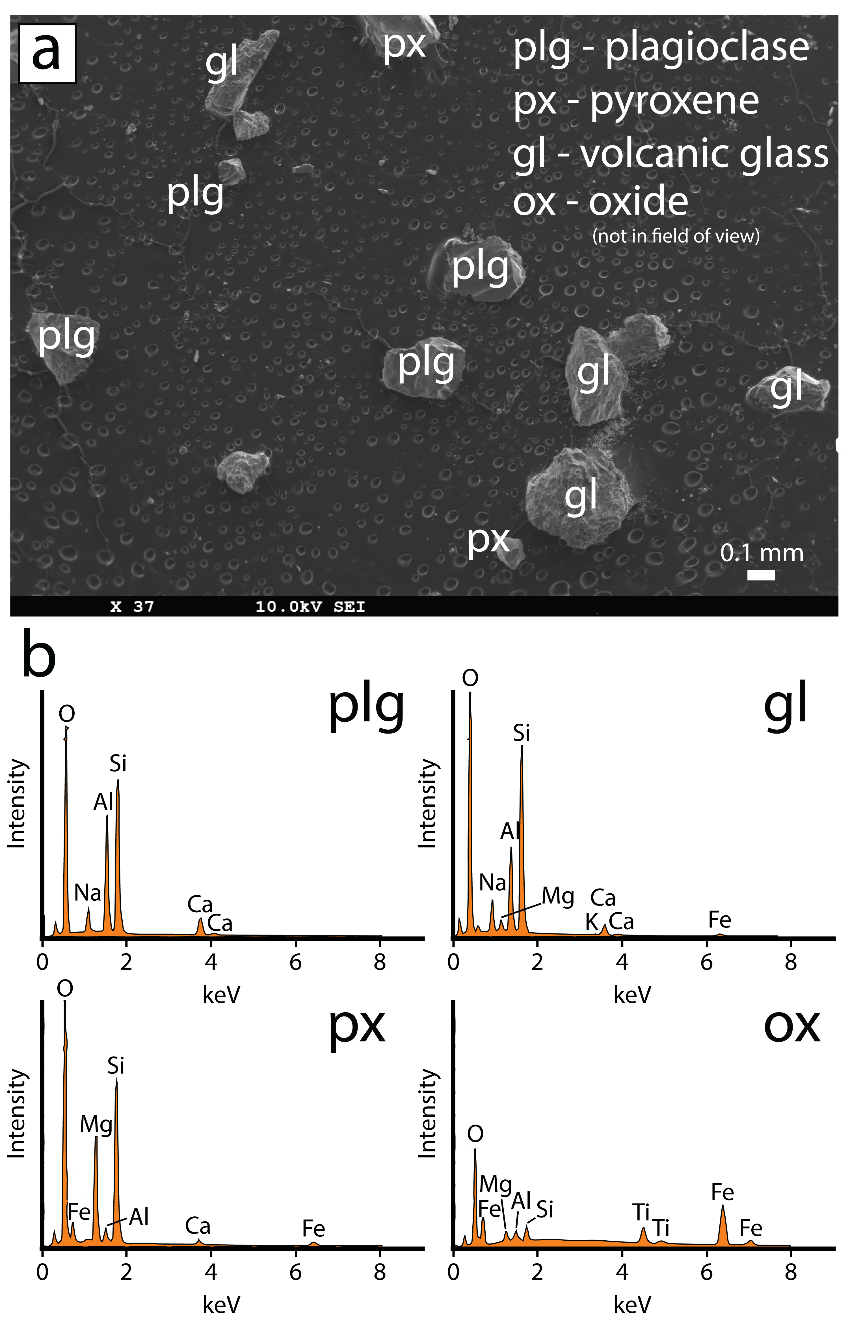


**Figure S1: Quantification of volcanic glass content using SEM-EDS particle identification and counting.** a) Example SEM Micrograph of sample T2 showing a randomly selected field of view at low magnification with identified particles. The micrograph was acquired at 10 kV and 1 nA. b) Representative EDS spectra of selected mineral phases from the tephra samples. All spectra were taken from carbon-coated samples mounted on carbon tape, which accounts for the carbon peak on the left.


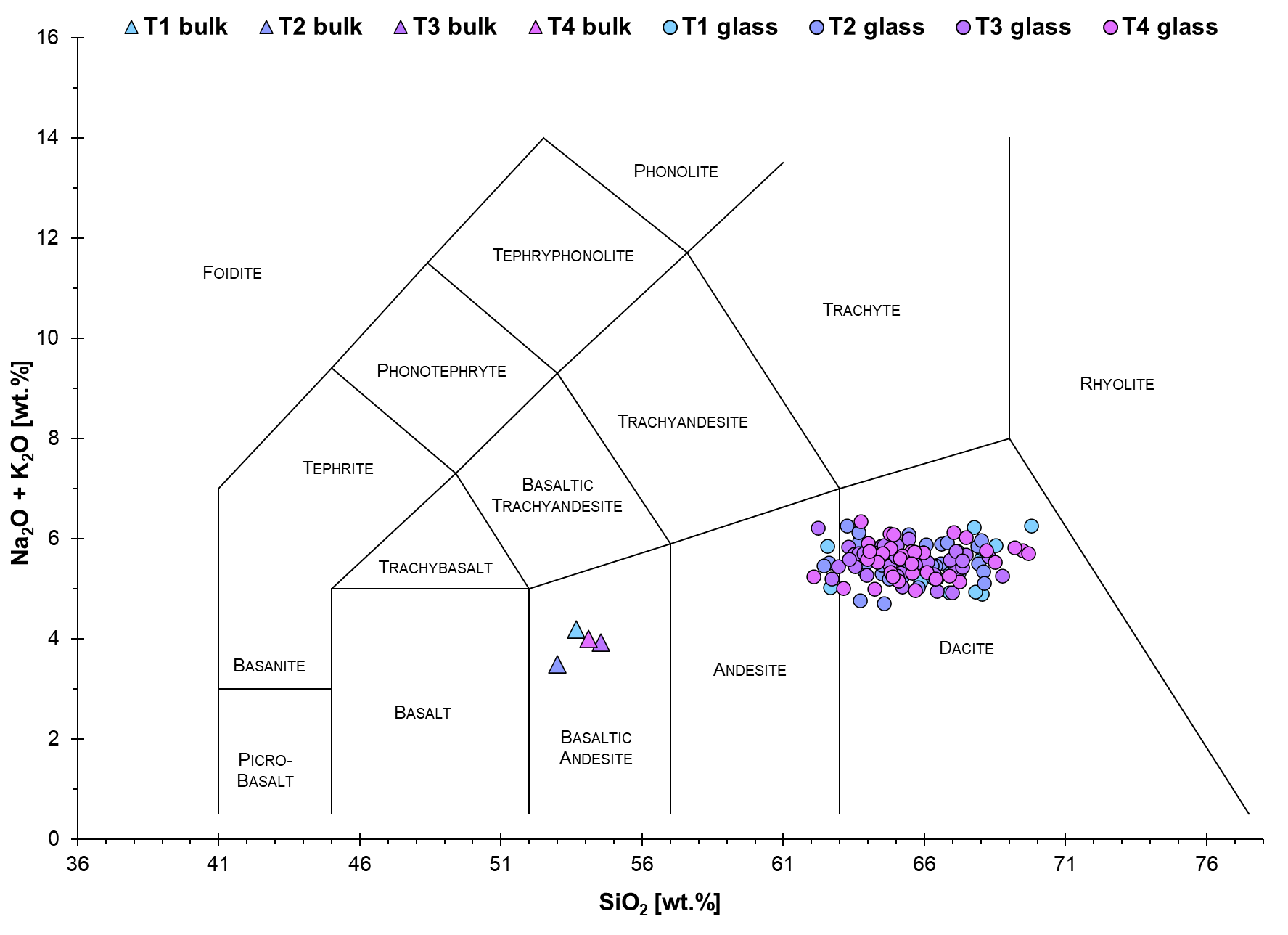


**Fig. S2: Total alkali silica (TAS) diagram with compositional data of the bulk tephra samples (triangles) obtained by XRF, and volcanic glass compositions within the tephra samples (circles) obtained by EPMA.** The colour scale is used to distinguish between samples.


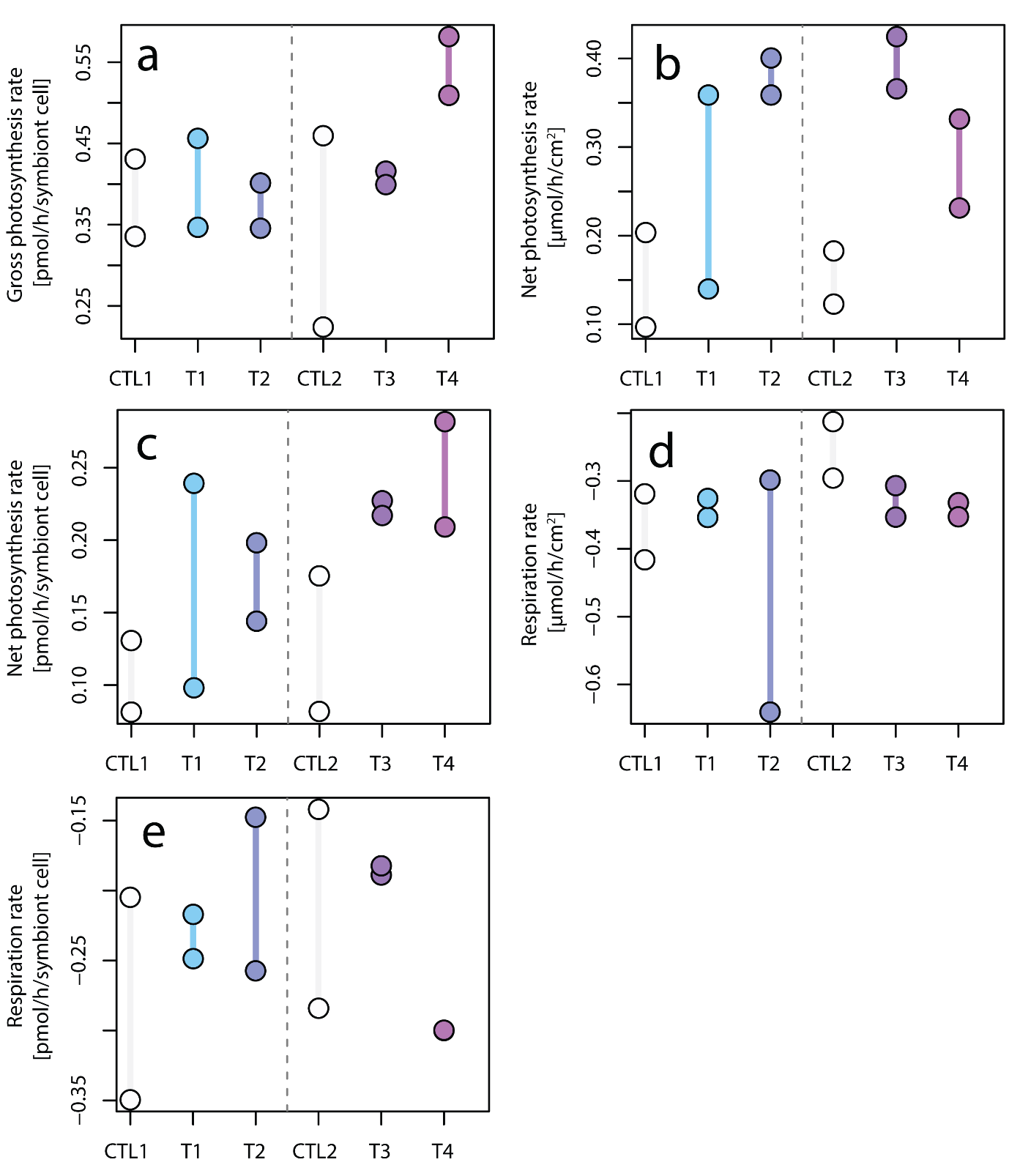


**Fig. S3: Photosynthesis and respiration rates (PR rates) of *S. pistillata* reared in control conditions (in white) and after 3 weeks of daily exposure to various tephra samples (1 g L^-1^).** PR rates are expressed as (a) gross oxygen production rate, (b) & (c) net photosynthesis rates, and (d) & (e) respiration rates. (a), (c) and (e) are normalized per symbiont cell, while (b) and (d) are normalized per skeletal surface area. Individual measurements are plotted as points (n = 2 per condition).


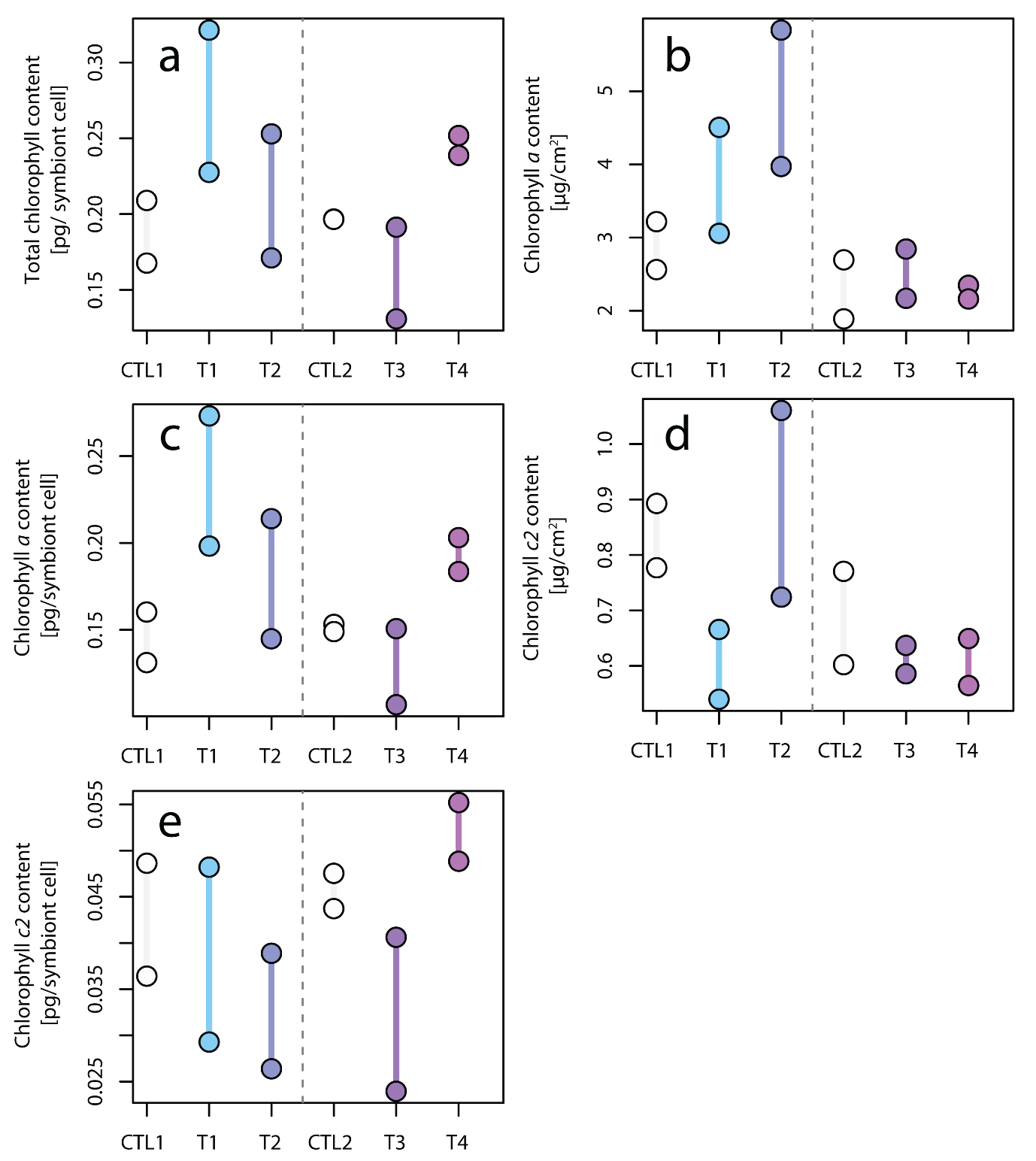


**Fig. S4: Chlorophyll content of *S. pistillata* reared in control conditions (in white) and after 3 weeks of daily exposure to various tephra samples (1 g L^-1^).** (a) Total chlorophyll content (*a* + *c2*), (b) & (c) chlorophyll *a* content, and (d) & (e) chlorophyll *c2* content. (b) and (d) are normalized per skeletal surface area, while (a), (c) and (e) are normalized per symbiont cell. Individual measurements are plotted as points (n = 2 per condition).

**Table S1: Quality control for XRF measurements on CRM BHVO-2.**

| **Oxide** | **BHVO-2** | **GeoRem preferred values** | |
| --- | --- | --- | --- |
|  | *[wt.%]* | *[wt.%]* | *Relative Deviation [%]* |
| **K_2_O** | 0.51 | 0.513 | 0.58 |
| **CaO** | 11.47 | 11.4 | 0.61 |
| **P_2_O_5_** | 0.27 | 0.2685 | 0.56 |
| **Na_2_O** | 2.22 | 2.219 | 0.05 |
| **MgO** | 7.26 | 7.257 | 0.04 |
| **TiO_2_** | 2.75 | 2.731 | 0.70 |
| **Fe2O_3_** | 12.36 | 12.39 | 0.24 |
| **MnO** | 0.17 | 0.169 | 0.59 |
| **SiO_2_** | 49.65 | 49.6 | 0.10 |
| **Al_2_O_3_** | 13.38 | 13.44 | 0.45 |
| **Cr_2_O_3_** | 0.04 | - | - |
| **NiO** | 0.02 | - | - |
| **H_2_O** | 0 | - | - |
| **CO_2_** | 0 | - | - |
| **Total** | 100.1 | 99.9875 |  |

**Tab. S2: Bulk chemical composition of tephra samples using XRF.** Major and minor elemental compositions are given as oxides in wt.%.

| **Oxide** | **T1** | **T2** | **T3** | **T4** |
| --- | --- | --- | --- | --- |
|  | *[wt.%]* | *[wt.%]* | *[wt.%]* | *[wt.%]* |
| **K_2_O** | 0.58 | 0.47 | 0.55 | 0.58 |
| **CaO** | 9.15 | 9.25 | 8.60 | 9.03 |
| **P_2_O_5_** | 0.12 | 0.11 | 0.14 | 0.13 |
| **Na_2_O** | 3.60 | 3.02 | 3.38 | 3.42 |
| **MgO** | 4.22 | 5.45 | 3.92 | 3.81 |
| **TiO_2_** | 0.91 | 1.01 | 0.92 | 0.92 |
| **Fe_2_O_3_** | 8.58 | 10.12 | 8.60 | 8.40 |
| **MnO** | 0.18 | 0.21 | 0.17 | 0.17 |
| **SiO_2_** | 53.65 | 52.98 | 54.52 | 54.11 |
| **Al_2_O_3_** | 18.58 | 17.38 | 18.68 | 18.97 |
| **Cr_2_O_3_** | 0.01 | 0.01 | 0.01 | 0.01 |
| **NiO** | 0.00 | 0.00 | 0.00 | 0.00 |
| **LOI** | 0.44 | -0.06 | 0.87 | 0.39 |
| **Total** | 100.02 | 99.95 | 100.34 | 99.93 |

**Tab. S3: Particle counting statistics of mineral phases identified in four tephra samples from St. Vincent.** Phase identification was based on the comparison of obtained SEM-EDS chemical compositions with published reference values from Weber et al. (2023).

| Mineral | Tephra sample | | | |
| --- | --- | --- | --- | --- |
|  | T1 | T2 | T3 | T4 |
| Volcanic Glass | 18 | 18 | 17 | 9 |
| Plagioclase | 19 | 20 | 26 | 25 |
| Pyroxene | 4 | 9 | 6 | 8 |
| Cristobalite | 1 | 1 | 3 | 1 |
| Oxide | 0 | 1 | 0 | 2 |
| Organics | 3 | 1 | 2 | 1 |
| Halite | 1 | 0 | 0 | 0 |
| Unidentified/Mixed | 6 | 15 | 3 | 4 |
| Total | 52 | 65 | 57 | 50 |
|  |  |  |  |  |
| Glass proportion [%] | 34.6 | 27.7 | 29.8 | 18.0 |
| Mineral proportion [%] | 65.4 | 72.3 | 70.2 | 82.0 |
| SE [%] | 6.6 | 5.6 | 6.1 | 5.4 |
| 95% confidence interval (CI) | ± 12.9 | ± 10.9 | ± 11.9 | ± 10.7 |

*CI = 1.96 * SE*

**Tab. S4: Chemical composition of volcanic glass within tephra samples using EMPA.** Major and minor elemental compositions are given as oxides in wt.%.

| **Oxide** | **T1** | | **T2** | | **T3** | | **T4** | |
| --- | --- | --- | --- | --- | --- | --- | --- | --- |
|  | *Median [wt.%]* | *SD [wt.%]* | *Median [wt.%]* | *SD [wt.%]* | *Median [wt.%]* | *SD [wt.%]* | *Median [wt.%]* | *SD [wt.%]* |
| **K_2_O** | 1.55 | 0.27 | 1.36 | 0.24 | 1.42 | 0.28 | 1.41 | 0.24 |
| **CaO** | 3.65 | 0.77 | 4.35 | 0.71 | 4.14 | 0.84 | 4.35 | 0.77 |
| **P_2_O_5_** | 0.36 | 0.06 | 0.33 | 0.08 | 0.32 | 0.08 | 0.32 | 0.07 |
| **Na_2_O** | 3.91 | 0.40 | 4.14 | 0.45 | 3.97 | 0.43 | 4.16 | 0.47 |
| **MgO** | 0.96 | 0.39 | 1.14 | 0.53 | 1.01 | 0.51 | 0.91 | 0.42 |
| **TiO_2_** | 1.04 | 0.14 | 1.03 | 0.17 | 1.05 | 0.18 | 1.09 | 0.23 |
| **FeO** | 6.44 | 0.84 | 6.56 | 1.07 | 6.58 | 0.99 | 6.27 | 1.25 |
| **MnO** | 0.18 | 0.03 | 0.17 | 0.05 | 0.18 | 0.04 | 0.17 | 0.04 |
| **SiO_2_** | 66.99 | 1.69 | 65.28 | 1.62 | 65.20 | 1.65 | 65.37 | 1.78 |
| **Al_2_O_3_** | 14.00 | 1.50 | 15.01 | 1.60 | 14.90 | 1.88 | 14.80 | 1.70 |
| **Total** | 99.51 | 0.53 | 99.58 | 0.87 | 99.50 | 0.88 | 99.68 | 0.69 |
|  | n = 25 [50] | | n = 32 [50] | | n = 37 [50] | | n=34 [50] | |

**Tab. S5: Quality control (accuracy and precision) for EMPA measurements on CRM VG-2.**

| **Oxide** | **VG-2  (USNM 111240/52)** | | | **GeoRem preferred values** | |
| --- | --- | --- | --- | --- | --- |
|  | *Median [wt.%]* | *SD [wt.%]* | *2RSD [%]* | *Median [wt.%]* | *Relative Deviation [%]* |
| **K_2_O** | 0.20 | 0.03 | 27.98 | 0.19 | 5.00 |
| **CaO** | 10.89 | 0.07 | 1.27 | 11.12 | 2.10 |
| **P_2_O_5_** | 0.20 | 0.05 | 43.91 | 0.20 | 0.50 |
| **Na_2_O** | 2.63 | 0.06 | 4.18 | 2.62 | 0.21 |
| **MgO** | 6.88 | 0.08 | 2.32 | 6.95 | 1.08 |
| **TiO_2_** | 1.79 | 0.04 | 4.32 | 1.85 | 3.03 |
| **FeO_tot_** | 11.50 | 0.10 | 1.71 | 12.06 | 4.66 |
| **MnO** | 0.17 | 0.01 | 16.60 | 0.22 | 21.14 |
| **SiO_2_** | 50.96 | 0.07 | 0.28 | 50.81 | 0.30 |
| **Al_2_O_3_** | 14.03 | 0.05 | 0.66 | 14.06 | 0.18 |
| **Total** | 99.26 | 0.13 | - | 100.08 | - |
|  | n = 4 | |  |  |  |

**Tab. S6: Mn concentrations [μg L^-1^] in seawater samples.** Each measurement point represents the Mn concentration measured in a designated beaker. Beakers were sampled at the start of each experimental week (Mondays). Week 1, 2 and 3 correspond to days 1, 8 and 15, respectively. The letter “C” next to a condition signifies the presence of corals in the beakers. “a” and “b” denote replicates of each condition. Control (Ctl1 & Ctl2) represents seawater blanks

| **Condition** | **Week 1** | | **Week 2** | | **Week 3** | |
| --- | --- | --- | --- | --- | --- | --- |
|  | *a* | *b* | *a* | *b* | *a* | *b* |
| **Ctl1** | 0.262 | 0.262 | 0.262 | 0.262 | 0.262 | 0.262 |
| **Ctl1 C** | 0.262 | 0.262 | 0.262 | 0.262 | 0.262 | 0.262 |
| **T1** | 2.929 | 0.888 | 1.300 | 0.763 | 0.662 | 0.443 |
| **T1 C** | 1.459 | 0.715 | 0.522 | 0.587 | 0.338 | 0.832 |
| **T2** | 1.517 | 1.465 | 0.703 | 1.569 | 0.887 | 1.111 |
| **T2 C** | 0.939 | 1.539 | 1.701 | 1.531 | 1.014 | 1.629 |
|  |  |  |  |  |  |  |
| **Ctl2** | 1.407 | 0.191 | 0.191 | 0.191 | 0.191 | 0.720 |
| **Ctl2 C** | 0.191 | 0.191 | 0.191 | 0.191 | 0.191 | 0.191 |
| **T3** | 2.328 | 2.845 | 2.097 | 5.360 | 1.942 | 1.654 |
| **T3 C** | 2.986 | 1.909 | 1.625 | 2.115 | 1.774 | 2.575 |
| **T4** | 13.025 | 12.348 | 13.963 | 12.496 | 14.143 | 12.842 |
| **T4 C** | 13.322 | 13.536 | 12.811 | 14.071 | 13.146 | 12.874 |
| *in purple: LOD, due to measured value < LOD* | | | |  |  |  |

**Tab. S7: Model fitting statistics for photophysiological parameters Φ_PSII_, ETR and P_gross_ plotted against seawater Mn concentrations.** a) Non-linear curve fit equation; b) estimated coefficients obtained *via* iterative least squares; c) and d) present statistical measures of model robustness. Model fit statistics were generated in OriginPro (OriginLab, 2024).

**a) Logarithmic model fit**: y = a – b * ln(x + c)

| **b) Coefficients** | | | **a** | | | | **b** | | | | **c** | | | |
| --- | --- | --- | --- | --- | --- | --- | --- | --- | --- | --- | --- | --- | --- | --- |
| *Parameter* | | | *Value* | *SE* | *t-Value* | *Prob>\|t\|* | *Value* | *SE* | *t-Value* | *Prob>\|t\|* | *Value* | *SE* | *t-Value* | *Prob>\|t\|* |
| Φ_PSII_ | Week 1 | | 0.37 | 0.01 | 37.81 | 2.35E-09 | -1.07E-03 | 0.01 | -0.14 | 0.90 | -0.20 | 1.54E-14 | -1.30E+13 | 4.16E-90 |
| Φ_PSII_ | Week 2 | | 0.45 | 0.01 | 39.14 | 1.85E-09 | -0.03 | 0.01 | -4.45 | 2.97E-03 | -0.16 | 0.04 | -3.68 | 7.84E-03 |
| Φ_PSII_ | Week 3 | | 0.50 | 0.01 | 59.19 | 1.03E-10 | -0.03 | 0.01 | -5.19 | 1.26E-03 | -0.20 | 4.52E-03 | -43.46 | 8.91E-10 |
| RLC | ETR | | 29.67 | 2.34 | 12.68 | 1.06E-03 | -2.47 | 1.06 | -2.34 | 0.10 | -0.19 | 0.01 | -15.18 | 6.21E-04 |
| P_gross_ | Average (Week 1- 3) | | 0.62 | 0.04 | 17.27 | 4.23E-04 | -0.04 | 0.01 | -2.56 | 0.08 | -0.19 | 0.01 | -15.72 | 5.60E-04 |
|  |  | |  |  |  |  |  |  |  |  |  |  |  |  |
| **c) Statistics** |  | |  |  |  |  |  | **d) ANOVA** |  |  |  |  |  |  |
| *Parameter* | *Number of Points* | *DF* | | *Reduced  χ^2^* | *Residual Sum  of Squares* | *R^2^ (COD)* |  | *Parameter* | | *DF* | *Sum of Squares* | *Mean Square* | *F Value* | *p-Value* |
| Φ_PSII_ | 10 | | 7 | 0.22 | 1.55 | 0.41 |  | Φ_PSII_ | Regression | 2 | 1.09 | 0.54 | 2.46 | 0.16 |
| Φ_PSII_ | 10 | | 7 | 0.52 | 3.63 | 0.90 |  | Φ_PSII_ | Regression | 2 | 33.46 | 16.73 | 32.25 | 2.94E-04 |
| Φ_PSII_ | 10 | | 7 | 0.42 | 2.92 | 0.95 |  | Φ_PSII_ | Regression | 2 | 59.07 | 29.54 | 70.78 | 2.27E-05 |
| ETR | 6 | | 3 | 3.99 | 11.97 | 0.78 |  | RLC | Regression | 2 | 43.43 | 21.71 | 5.44 | 0.10 |
| P_gross_ | 6 | | 3 | 2.32 | 6.96 | 0.73 |  | P_gross_ | Regression | 2 | 19.16 | 9.58 | 4.13 | 0.14 |
| *SE - Standard Error; DF - Degrees of Freedom; COD -Coefficient of Determination* | | | | | |  |  |  |  |  |  |  |  |  |

OriginLab. (2024). *OriginPro (Version 2024 Academic)*. In OriginLab Corporation. <https://www.originlab.com>

Weber, G., Blundy, J., Barclay, J., Pyle, D. M., Cole, P., Frey, H., Manon, M., Davies, B. V., & Cashman, K. (2023). Petrology of the 2020–21 effusive to explosive eruption of La Soufrière Volcano, St Vincent: insights into plumbing system architecture and magma assembly mechanism. *Geological Society, London, Special Publications*, *539*(1), 171-200. <https://doi.org/10.1144/sp539-2022-177>
